# An electronic transition-based bare bones particle swarm optimization algorithm for high dimensional optimization problems

**DOI:** 10.1371/journal.pone.0271925

**Published:** 2022-07-25

**Authors:** Hao Tian, Jia Guo, Haiyang Xiao, Ke Yan, Yuji Sato

**Affiliations:** 1 School of Information and Communication Engineering, Hubei University of Economics, Wuhan, China; 2 Smart Business Department of China Construction Third Engineering Bureau Installation Engineering Co., Ltd., Wuhan, China; 3 Faculty of Computer and Information Sciences, Hosei University, Tokyo, Japan; Torrens University Australia, AUSTRALIA

## Abstract

An electronic transition-based bare bones particle swarm optimization (ETBBPSO) algorithm is proposed in this paper. The ETBBPSO is designed to present high precision results for high dimensional single-objective optimization problems. Particles in the ETBBPSO are divided into different orbits. A transition operator is proposed to enhance the global search ability of ETBBPSO. The transition behavior of particles gives the swarm more chance to escape from local minimums. In addition, an orbit merge operator is proposed in this paper. An orbit with low search ability will be merged by an orbit with high search ability. Extensive experiments with CEC2014 and CEC2020 are evaluated with ETBBPSO. Four famous population-based algorithms are also selected in the control group. Experimental results prove that ETBBPSO can present high precision results for high dimensional single-objective optimization problems.

## Introduction

An electron transition is essentially an energy change of electrons in the particles that make up matter. According to the principle of conservation of energy, the outer electrons of a particle absorb energy as they move from a lower to a higher energy level, and release energy as they move from a higher to a lower energy level. The energy is the absolute value of the difference between the energies of the two energy levels. In this paper, we use particles to simulated the electronic transition behavior to solve high dimensional optimization problems. Particles are designed to search in different orbits. The particles on the worse-positioned orbits have the opportunity to make a transition to the better-positioned orbits.

Optimization problems appear everywhere in our daily life. Whenever we want to make a choice, we believe the option is better. In numerical optimization problems, the numerous global optimization (GO) problems is often described in Eq 1:

$$\begin{array}{c} \begin{array}{c} f:\;\;\;\;\;X\to R\\ x^{*}\in X\\ f\left(x^{*}\right)\leq f\left(X\right) \end{array} \end{array}$$ (1)

where $X\subset R^{D}$ is a nonempty compact set that contains all feasible solutions, *D* is the dimension of the problem, *f* is a real valued objective function, x* is the theoretical optimal solution [1]. The purpose of an optimization algorithms is finding the x*, even the objective functions maybe non-convex, multimodal, or badly scaled [2].

To solve numerical optimization problems, population-based methods like genetic algorithms (GA) [3], differential evolution (DE) [4], particle swarm optimization (PSO) [5] ant colony (AC) [6], walf pack algorithm [7] are proposed. Among these methods, PSO is famous for fast convergence and easy applying. PSO algorithms are widely applied in engineering like route planning [8, 9], data clustering [10, 11], feature selection [12, 13], image segmentation [14, 15], power system [16, 17], engineering areas [18–20], and so on.

The class PSO algorithm is inspired by the social behavior of fish and birds. Particles begin searching from random solutions and aim at the solution which can minimize the target problem. In PSO, each particle represents a solution of the target problem. In function optimizations, each particle retains follow attributes: velocity, represents how fast a particle is moving in the search space; current fitness value, represents the function value at current position; current position, represents the coordinate at this generation; personal best value, represents the best function value across all generations; personal best position, represents the coordinate of the personal best value. The behavior of the particles is controlled by many parameters, so to achieve the best performance researchers need to adjust parameters for every specific problem. Also, with the developing of technology, optimization problems become high dimensional and multimodal. Traditional PSO methods sometimes difficult to provide high precision results. For some complicate prblems, PSO methods are easily fall into local minimal and leading to unacceptable results.

Researchers tried to improve the performance of PSO by combining different strategies. In 2016, Pornsing [21] propsoed a self-adaptive strategy to imoprove the search ability of PSO.In 2017, Chen proposed a new biogeography-based learning strategy for PSO [22]. In 2018, Xu proposed a novel chaotic PSO for combination optimization problems [23]. In 2018, Tian proposed a chaos-based initialization strategy and robust update mechanisms for PSO [24]. In 2019, Ghasemi proposed a new parameter control strategy to enhance the search ability of PSO [25]. In 2019, Xu [26] combined the quantum behavior with PSO and achieved better search ability. In 2021, Yamanaka tried to improve the performance PSO by introducing the new concept of particle clustering [27].

Bare bones PSO (BBPSO) [28] is a simple type of PSO. With the cancellation of the velocity term, BBPSO can solve different types of optimization problems without any parameters. In 2017, Guo [29] combined a pair-wise strategy with BBPSO (PBBPSO). Particles change information with a pair-particle during iterations. In addition, three particles are placed in one local group in hierarchical BBPSO (HBBPSO) [30]. Three particles form two different spatial structures to handle different optimization problems. On the other hand, Guo [31] proposed a dynamic local search strategy to enhance the local search ability of BBPSO. In 2018, Guo [32] developed a dynamic allocation operator for BBPSO. In 2019, Guo [33] proposed a fission-fusion strategy for BBPSO. In 2020, Guo [34] proposed a novel BBPSO based method for traveling salesman problem (TSP). Proposed method can present high precision for TSPs.

The rest of this paper is organized as follows: Section 2 introduces the proposed method; Section 3 introduces the numerical experiments; Section 4 presents the conclusions of this paper.

## Materials and methods

The electronic transition-based bare bones particle swarm optimization (ETBBPSO) algorithm is proposed in this section.

### Particle swarm explosion

The initialization of ETBBPSO is called particle swarm explosion (PSE). In PSE, particles are randomly dispersed into the search space. Then, the personal best position and the personal best value of every particle will be calculated. The global best position and the global best value of the particle swarm will be recorded.

### Dynamic particle grouping

In ETBBPSO, particles will be assigned to different orbits. During the evolutionary process, particles in orbits play two different roles: the core and the satellite. Each orbits contains one core and several satellites. The number of satellites can be zero. Different evolutionary strategies are applied to different roles. A core particle aims at searching around the global best particles and Enhancing the global search capability of the orbit. The candidate position of a core particle is selected by Eq 2.

$$\begin{array}{c} \begin{array}{c} \alpha =\frac{\left(pbest\left(core^{t}\right)+Gbest^{t}\right)}{2}\\ \beta \;=|pbest\left(core^{t}\right)-Gbest^{t}|\\ pbest{\left(core^{t+1}\right)}_{candidate}=GauDis\left(\alpha ,\beta \right) \end{array} \end{array}$$ (2)

where the *pbest*(*core*^*t*^) is the personal best position of the core particle in (*t*)th generation, *Gbest*^*t*^ is the personal best position of the global best particle in (*t*)th generation, *pbest*(*core*^*t*+1^)_*c*_ *andidate* is the candidate new position for main particle in (*t* + 1)th generation, *GauDis*(*α*, *β*) is the Gaussian distribution with a mean *α* and a standard deviation *β*.

A satellite particle aims at searching around the core of the orbit and implementing a local search. The candidate position of a satellite particle is selected by Eq 3.

$$\begin{array}{c} \begin{array}{c} \gamma =\frac{(pbest\left(core^{t}\right)+\left(pbest\left(satellite^{t}\right)\right)}{2}\\ \delta \;=|pbest(core^{t}-\left(pbest\left(satellite^{t}\right)\right)|\\ pbest{\left(satellite^{t+1}\right)}_{candidate}=GauDis\left(\gamma ,\delta \right) \end{array} \end{array}$$ (3)

where the *pbest*(*core*^*t*^) is the personal best position of the core particle in (*t*)th generation, where the *pbest*(*satellite*^*t*^) is the personal best position of the satellite particle in (*t*)th generation, *pbest*(*satellite*^*t*^) is the personal best position of the global best particle in (*t*)th generation, *pbest*(*satellite*^*t*+1^)_*c*_ *andidate* is the candidate new position for the satellite particle in (*t* + 1)th generation, *GauDis*(*γ*, *δ*) is the Gaussian distribution with a mean *γ* and a standard deviation *δ*.

A dynamic particle grouping (DPG) strategy is used to divide the particle swarm into different orbits. At the beginning of DPG, the particle *x*_1_ is selected as the core of the first Orbit. Then the next particle is selected to compare with the previous core. If the selected particle is better than the previous core, a new orbit will be created and the selected particle will be the core of the new orbit. Otherwise the particle will be a satellite of the original orbit. Then this process will be repeated until all particles have been assigned to orbits. The pseudo code of DPG is shown in Algorithm 1.

**Algorithm 1** Dynamic Particle Grouping

**Require:** Max generation time, *MIT*

**Require:** Test function, *F*

**Require:** Search Space, *R*

**Require:** Number of particle, *n*

**Require:** *Pbest_value*

**Require:** *Pbest_position*

**Require:** *Gbest_value*

**Require:** *Gbest_position*

**Require:** *No*

**Require:** *t*

1: **while** *t* < *MIT* **do**

2:  **if** *NO* == 1 **then**

3:   *t* = *t* + 1

4:   Select the first particle *x*_1_ as the core for *Orbit*(*NO*)

5:   *CurrentCore* = *x*_*k*_

6:   **for** *i* in range (2, *n*) **do**

7:    **if** *pb*_*i*_ < *pb*_*CurrentCore*_ **then**

8:     Select a new position for *x*_*i*_ according to Eq 2

9:     Create a new Orbit, *NO* = *NO* + 1

10:     *CurrentCore* = *x*_*i*_

11:    **else**

12:     Make *x*_*i*_ a satellite of *CurrentCore*, belonging to *Orbit*(*NO*)

13:     Select a new position for *x*_*i*_ according to Eq 3

14:    **end if**

15:   **end for**

16:   Update *Pbest_value*, *Pbest_position*, *Gbest_value*,*Gbest position*

17:   In each Orbit, make the particle with a smallest *Pbest_value* be the new core

18:  **end if**

19: **end while**

### Particle transition

To enhance the local search ability of the top orbit, the particle transition operator (PTO) is proposed. All orbits will be ranked according to the personal best value of their cores. Then all particles in the second best orbit will transit to the best orbit. By doing this the best orbit will gather more and more particles to obtain stronger local search ability. Other orbits still have change to do global search and replace the best orbit. The pseudo code of the PTO is shown in Algorithm 2.

**Algorithm 2** Particle transition operator

**Require:** Max generation time, *MIT*

**Require:** Test function, *F*

**Require:** Search Space, *R*

**Require:** Number of particle, *n*

**Require:** *Pbest_value*

**Require:** *Pbest_position*

**Require:** *Gbest_value*

**Require:** *Gbest_position*

**Require:** *NO*, *t*

**Require:** *Orbit*

1: **while** *t* < *MIT* **do**

2:  **if** *NO* > 1 **then**

3:   *t* = *t* + 1

4:   Rank all Orbits according the personal best values of their cores

5:   A core with a smaller personal best value is a better core, its corresponding Orbit is the better Orbit

6:   Merge the first and second best Orbits

7:   Select a new position for all cores according to Eq 2

8:   Select a new position for all satellites according to Eq 3

9:   *NO* = *NO* − 1

10:   Update *Pbest_value*,*Pbest_position*, *Gbest_value*,*Gbest_position*

11:  **end if**

12: **end while**

### Console

**Fig 1:**
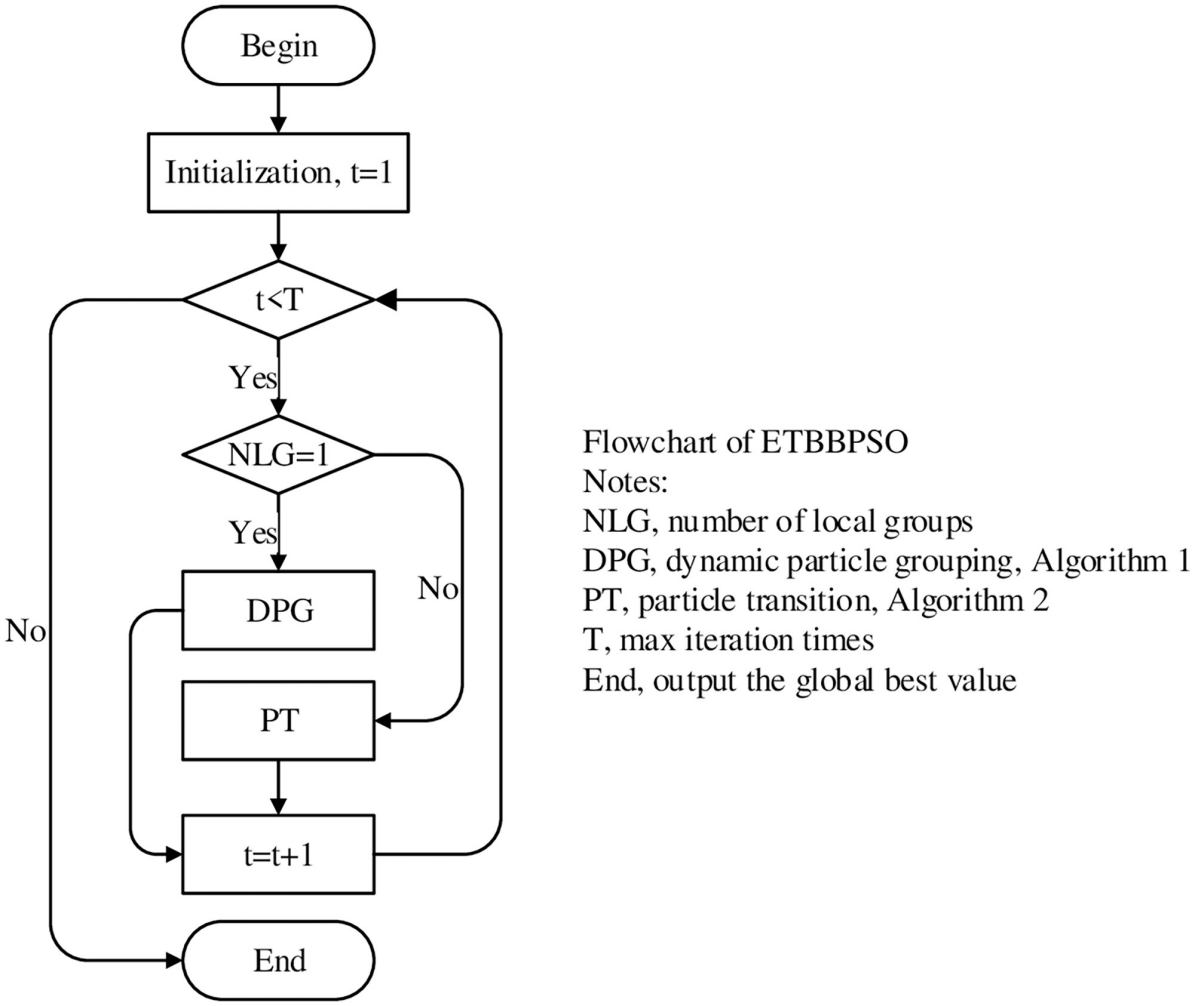
The flowchart of ETBBPSO.

The DPG and the PTO collaborate to balance the local and global search. The DPG adaptive grouping strategy is applied so that the internal structure of the particle swarm can change with the change of the target problem. The PTO will merge the top two orbits. By doing this, the distribution of orbits enables the particle swarm to take into account the global search capability while enhancing the local search capability in specific regions. The overall process of ETBBPSO is shown in Algorithm 3. The flowchart of the ETBBPSO is shown in Fig 1.

**Algorithm 3** Console

**Require:** Max generation time, *MIT*

**Require:** Test function, *F*

**Require:** Search Space, *R*

**Require:** Number of particle, *n*

**Require:** *Pbest_value*, *Pbest_position*, *Gbest_value*, *Gbest_position*

**Require:** *NO*, *t*, *Orbit*

1: Run PSE

2: **while** *t* < *MIT* **do**

3:  **if** *NO* == 1 **then**

4:   Run Algorithm 1

5:  **end if**

6:  **if** *NO* > 1 **then**

7:   Run Algorithm 2

8:  **end if**

9: **end while**

## Results

### Experiments with CEC2014

In order to conduct a fair and comprehensive comparison, the CEC2014 benchmark functions are selected in experiments. Four famous population based methods PBBPSO [29], DABBPSO [32], DLS-BBPSO [31], and FBBPSO [33] are selected in the control group. PBBPSO conducts a novel paired evolution strategy and has shown reliable performance in single modal and multi-modal optimization problems. DABBPSO integrates the scattering and ordering of particle swarms. DLS-BBPSO carries out a dynamic local search operator and show powerful ability in single-objective optimization problems. FBBPSO is the state-of-the-art method based on Bare-bones PSO and has shown great performance and stability on CEC2014. To test the extreme optimization capability of ETBBPSO, experiments are conducted in 100-dimension, max generation time is 1.00E+4, population size for all algorithms is 100. Details of benchmark functions can be found in Table 1. All code are written in Matlab R2020b.

**Table 1:** Experimental functions, the CEC 2014 benchmark functions, the search range for each function is (-100,100), the dimension is 100.

Types	Function Name	Dimension	Search Range	Theoretically Minimum
Unimodal Functions	*f*_1_ = Rotaten High Conditioned Elliptic Function	100	(-100,100)	100
	*f*_2_ = Rotated Bent Cigar Function	100	(-100,100)	200
	*f*_3_ = Rotated Discus Function	100	(-100,100)	300
Simple Multimodal Functions	*f*_4_ = Shifted and Rotated Rosenbrock’s Function	100	(-100,100)	400
	*f*_5_ = Shifted and Rotated ACKLEY’s Function	100	(-100,100)	500
	*f*_6_ = Shifted and Rotated Weierstrass’s Function	100	(-100,100)	600
	*f*_7_ = Shifted and Rotated Griewank’s Function	100	(-100,100)	700
	*f*_8_ = Shifted Rastrigin’s Function	100	(-100,100)	800
	*f*_9_ = Shifted and Rotated Rastrigin’s Function	100	(-100,100)	900
	*f*_10_ = Shifted Schwefel’s Function	100	(-100,100)	1000
	*f*_11_ = Shifted and Rotated Schwefel’s Function	100	(-100,100)	1100
	*f*_12_ = Shifted and Rotated Katsure Function	100	(-100,100)	1200
	*f*_13_ = Shifted and Rotated HappyCat Function	100	(-100,100)	1300
	*f*_14_ = Shifted and Rotated HGBat Function	100	(-100,100)	1400
	*f*_15_ = Shifted and Rotated Expanded	100	(-100,100)	1500
	Griewank’s plus Rosenbrock’s Function	100	(-100,100)	
	*f*_16_ = Shifted and Rotated	100	(-100,100)	1600
	Expanded Scaffer’s F6 Function	100	(-100,100)	
Hybrid Functions	*f*_17_ = Hybrid Function 1 (N = 3)	100	(-100,100)	1700
	*f*_18_ = Hybrid Function 2 (N = 3)	100	(-100,100)	1800
	*f*_19_ = Hybrid Function 3 (N = 4)	100	(-100,100)	1900
	*f*_20_ = Hybrid Function 4 (N = 4)	100	(-100,100)	2000
	*f*_21_ = Hybrid Function 5 (N = 5)	100	(-100,100)	2100
	*f*_22_ = Hybrid Function 6 (N = 5)	100	(-100,100)	2200
Composition Functions	*f*_23_ = Composition Function 1 (N = 5)	100	(-100,100)	2300
	*f*_24_ = Composition Function 2 (N = 3)	100	(-100,100)	2400
	*f*_25_ = Composition Function 3 (N = 3)	100	(-100,100)	2500
	*f*_26_ = Composition Function 4 (N = 5)	100	(-100,100)	2600
	*f*_27_ = Composition Function 5 (N = 5)	100	(-100,100)	2700
	*f*_28_ = Composition Function 6 (N = 5)	100	(-100,100)	2800
	*f*_29_ = Composition Function 7 (N = 3)	100	(-100,100)	2900
	*f*_30_ = Composition Function 8 (N = 3)	100	(-100,100)	3000

#### Experimental results and discussion

Experimental results are shown in Tables 2 and 3. *Mean* is the mean calculation error (CE) from 31 independent runs. CE is defined as |*GlobalBestValue* − *TheoreticallyMinimum*|. *Std* is the standard deviation of the 31 independent runs, *Rank* is the ranking results of the six algorithms. The Wilcoxon rank sum test is also implemented and average rank results are shown in Table 4.

**Table 2:** Experimental results, CE of PSO, PBBPSO, DA-BBPSO, DLS-BBPSO, FBBPSO and ETBBPSO for *f*_1_−*f*_15_. Mean is the mean valut from 31 independent runs, STD is the standard deviation of the 31 runs, Rank is the rank of 6 algorithms.

Function Number	Data Tpye	PSO	PBBPSO	DA-BBPSO	DLS-BBPSO	FBBPSO	ETBBPSO
*f*_1_	Mean	1.454E+08	4.725E+07	4.253E+07	4.872E+07	5.172E+07	4.339E+07
	Std	6.297E+07	1.608E+07	1.514E+07	1.505E+07	1.934E+07	1.797E+07
	Rank	6	3	1	4	5	2
*f*_2_	Mean	9.363E+06	2.879E+04	5.013E+04	4.562E+04	3.781E+04	4.392E+04
	Std	5.200E+07	4.923E+04	5.615E+04	4.438E+04	4.272E+04	5.973E+04
	Rank	6	1	5	4	2	3
*f*_3_	Mean	6.772E+03	2.103E+04	1.736E+04	1.647E+04	1.893E+04	1.631E+04
	Std	3.541E+03	1.666E+04	1.462E+04	1.391E+04	1.216E+04	1.052E+04
	Rank	1	6	4	3	5	2
*f*_4_	Mean	5.351E+02	1.356E+02	1.470E+02	1.282E+02	1.551E+02	1.624E+02
	Std	1.145E+02	4.452E+01	5.656E+01	4.246E+01	4.468E+01	4.725E+01
	Rank	6	2	3	1	4	5
*f*_5_	Mean	2.127E+01	2.131E+01	2.131E+01	2.131E+01	2.132E+01	2.131E+01
	Std	4.477E-02	3.207E-02	2.341E-02	2.662E-02	2.290E-02	2.535E-02
	Rank	1	3	4	5	6	2
*f*_6_	Mean	7.908E+01	1.564E+02	1.517E+02	1.233E+02	1.055E+02	1.036E+02
	Std	6.381E+00	1.074E+01	1.765E+01	3.031E+01	1.764E+01	1.928E+01
	Rank	1	6	5	4	3	2
*f*_7_	Mean	4.081E-03	4.133E-03	1.987E-03	3.259E-03	4.606E-03	2.780E-03
	Std	6.490E-03	5.486E-03	4.380E-03	5.736E-03	7.290E-03	5.494E-03
	Rank	4	5	1	3	6	2
*f*_8_	Mean	1.340E+02	3.205E+02	3.704E+02	3.254E+02	3.281E+02	3.407E+02
	Std	1.807E+01	6.020E+01	5.821E+01	4.384E+01	4.966E+01	4.927E+01
	Rank	1	2	6	3	4	5
*f*_9_	Mean	3.573E+02	9.789E+02	1.006E+03	9.273E+02	1.059E+03	9.322E+02
	Std	5.343E+01	1.442E+02	1.404E+02	1.586E+02	1.539E+02	1.740E+02
	Rank	1	4	5	2	6	3
*f*_10_	Mean	3.705E+03	6.341E+03	8.020E+03	6.390E+03	6.642E+03	6.543E+03
	Std	7.200E+02	8.816E+02	2.003E+03	8.226E+02	9.828E+02	8.886E+02
	Rank	1	2	6	3	5	4
*f*_11_	Mean	1.492E+04	3.173E+04	3.249E+04	2.881E+04	2.346E+04	2.542E+04
	Std	2.896E+03	3.209E+03	3.138E+03	7.556E+03	8.871E+03	7.969E+03
	Rank	1	5	6	4	2	3
*f*_12_	Mean	3.399E+00	3.987E+00	4.040E+00	4.015E+00	3.960E+00	3.901E+00
	Std	3.983E-01	2.166E-01	1.733E-01	2.399E-01	4.298E-01	6.487E-01
	Rank	1	4	6	5	3	2
*f*_13_	Mean	6.861E-01	7.117E-01	7.211E-01	7.375E-01	6.858E-01	7.059E-01
	Std	5.123E-02	8.652E-02	1.013E-01	9.950E-02	8.404E-02	8.266E-02
	Rank	2	4	5	6	1	3
*f*_14_	Mean	3.855E-01	4.972E-01	5.907E-01	5.608E-01	6.102E-01	5.452E-01
	Std	1.500E-01	2.573E-01	3.229E-01	2.760E-01	2.894E-01	2.632E-01
	Rank	1	2	5	4	6	3
*f*_15_	Mean	6.745E+01	6.357E+01	7.252E+01	5.186E+01	6.924E+01	6.724E+01
	Std	1.249E+01	1.804E+01	1.858E+01	1.901E+01	1.919E+01	2.413E+01
	Rank	4	2	6	1	5	3

**Table 3:** Experimental Results, CE of PSO, PBBPSO, DA-BBPSO, DLS-BBPSO, FBBPSO and ETBBPSO for *f*_16_−*f*_30_. Mean is the mean valut from 31 independent runs, STD is the standard deviation of the 31 runs, Rank is the rank of 6 algorithms. Alvrage rank point is at the bottom of the table.

Function Number	Data Tpye	PSO	PBBPSO	DA-BBPSO	DLS-BBPSO	FBBPSO	ETBBPSO
*f*_16_	Mean	4.574E+01	4.741E+01	4.715E+01	4.712E+01	4.665E+01	4.678E+01
	Std	4.751E-01	9.261E-01	9.833E-01	8.539E-01	9.872E-01	9.423E-01
	Rank	1	6	5	4	2	3
*f*_17_	Mean	1.497E+07	9.276E+06	7.522E+06	8.617E+06	9.513E+06	7.641E+06
	Std	6.872E+06	2.908E+06	3.370E+06	4.707E+06	6.360E+06	3.086E+06
	Rank	6	4	1	3	5	2
*f*_18_	Mean	1.474E+05	9.621E+03	1.303E+04	7.654E+03	1.132E+04	1.008E+04
	Std	8.087E+05	1.179E+04	2.912E+04	7.838E+03	1.296E+04	1.284E+04
	Rank	6	2	5	1	4	3
*f*_19_	Mean	1.679E+02	1.088E+02	1.080E+02	1.135E+02	1.072E+02	1.065E+02
	Std	1.780E+01	4.609E+01	3.587E+01	5.465E+01	4.175E+01	4.431E+01
	Rank	6	4	3	5	2	1
*f*_20_	Mean	9.281E+03	2.410E+05	1.920E+05	2.112E+05	1.614E+05	1.443E+05
	Std	2.844E+03	2.549E+05	1.207E+05	1.428E+05	1.337E+05	8.422E+04
	Rank	1	6	4	5	3	2
*f*_21_	Mean	6.073E+06	4.210E+06	4.481E+06	5.010E+06	5.283E+06	4.672E+06
	Std	3.942E+06	1.955E+06	2.218E+06	2.894E+06	2.809E+06	2.093E+06
	Rank	6	1	2	4	5	3
*f*_22_	Mean	2.157E+03	5.133E+03	3.902E+03	5.345E+03	3.721E+03	4.044E+03
	Std	5.585E+02	1.452E+03	1.231E+03	1.376E+03	6.787E+02	1.194E+03
	Rank	1	5	3	6	2	4
*f*_23_	Mean	3.531E+02	3.472E+02	3.450E+02	3.450E+02	3.450E+02	3.450E+02
	Std	1.536E+00	1.215E+01	1.036E-05	2.707E-05	4.445E-05	6.985E-06
	Rank	6	5	2	3	4	1
*f*_24_	Mean	3.850E+02	3.889E+02	3.949E+02	3.901E+02	3.925E+02	3.892E+02
	Std	4.407E+00	5.792E+00	7.376E+00	4.694E+00	6.482E+00	7.166E+00
	Rank	1	2	6	4	5	3
*f*_25_	Mean	2.807E+02	2.046E+02	2.046E+02	2.045E+02	2.048E+02	2.043E+02
	Std	1.254E+01	1.043E+00	8.726E-01	8.597E-01	1.138E+00	9.194E-01
	Rank	6	4	3	2	5	1
*f*_26_	Mean	2.119E+02	1.007E+02	1.007E+02	1.007E+02	1.007E+02	1.007E+02
	Std	5.258E+01	9.343E-02	8.228E-02	7.951E-02	7.457E-02	6.422E-02
	Rank	6	3	2	4	1	5
*f*_27_	Mean	2.242E+03	4.229E+03	3.911E+03	3.674E+03	3.232E+03	3.214E+03
	Std	1.554E+02	4.651E+02	6.673E+02	6.689E+02	4.774E+02	4.074E+02
	Rank	1	6	5	4	3	2
*f*_28_	Mean	4.937E+03	5.430E+02	5.490E+02	5.587E+02	5.461E+02	5.448E+02
	Std	1.227E+03	5.380E+01	7.079E+01	7.318E+01	3.959E+01	6.800E+01
	Rank	6	1	4	5	3	2
*f*_29_	Mean	3.409E+03	2.664E+02	2.739E+02	2.777E+02	2.941E+02	2.947E+02
	Std	7.679E+02	2.396E+01	3.576E+01	4.246E+01	5.769E+01	6.961E+01
	Rank	6	1	2	3	4	5
*f*_30_	Mean	7.110E+04	3.965E+03	4.101E+03	3.600E+03	3.990E+03	3.895E+03
	Std	3.459E+04	9.916E+02	9.365E+02	6.542E+02	1.013E+03	8.328E+02
	Rank	6	3	5	1	4	2
Average Rank		3.40	3.47	4.00	3.53	3.83	2.77

**Table 4:** Parameters of the CEC2020 test.

Dimension	20
Populatiuon size	100
Max iteration times	10000
Independent runs	31
Search Range	[-100,100]
Control Group	FBBPSO and BBPSO

Numerical analyses are listed below: In *f*19, *f*23, *f*25, the result of ETBBPSO is ranked second among the six algorithms. In *f*1, *f*3, *f*5 − 7, *f*12, *f*17, *f*20, *f*27, *f*28, *f*30, the result of ETBBPSO is ranked second among the six algorithms. In *f*2, *f*9, *f*11, *f*13−16, *f*18, *f*21, *f*24, the result of ETBBPSO is ranked third among the six algorithms. It can be seen that ETBBPSO can present the top three results in 24 test functions. This proves that B is able to give an efficient optimization solution for most problems. It also proves that the electronic transition strategy can provide acceptable solutions for different problems. In *f*10 and *f*22, the result of ETBBPSO is ranked fourth among the six algorithms. In *f*4, *f*8, *f*26, *f*29, the result of ETBBPSO is ranked fifth among the six algorithms. These results suggest that ETBBPSO search ability is easily bounded in the face of such problems, and this is a major direction for future research. It is worth noting that ETBBPSO never finished last in the ranking test, which proves that ETBBPSO does not give extremely bad results even when faced with problems that it is not very good at handling. A ranking competition is designed for every test function. The algorithm presents the best results will get 1 point, presents the second-best results will get 2 points, presents the third-best results will get 3 points, presents the fourth-best results will get 4 points, presents the fifth-best results will get 5 points, presents the worst results will get 6 points. The mean ranking results are shown at the bottom of Table 2. ETBBPSO shows the best results in the 100-dimension test. This is mainly because DPG is able to divide the particle swarm into different orbits. The whole partitioning process is self-controlled by the algorithm and does not require any parameters. Then, PTO enhances the local search ability of particle swarm while taking into account the global search ability, making it more possible for the particle swarm to escape from the local optimum.

Evolutionary curves across iterations are shown in Figs 2 to 31. The horizontal axis represents the number of iterations, vertical axes represents CEs.

**Fig 2:**
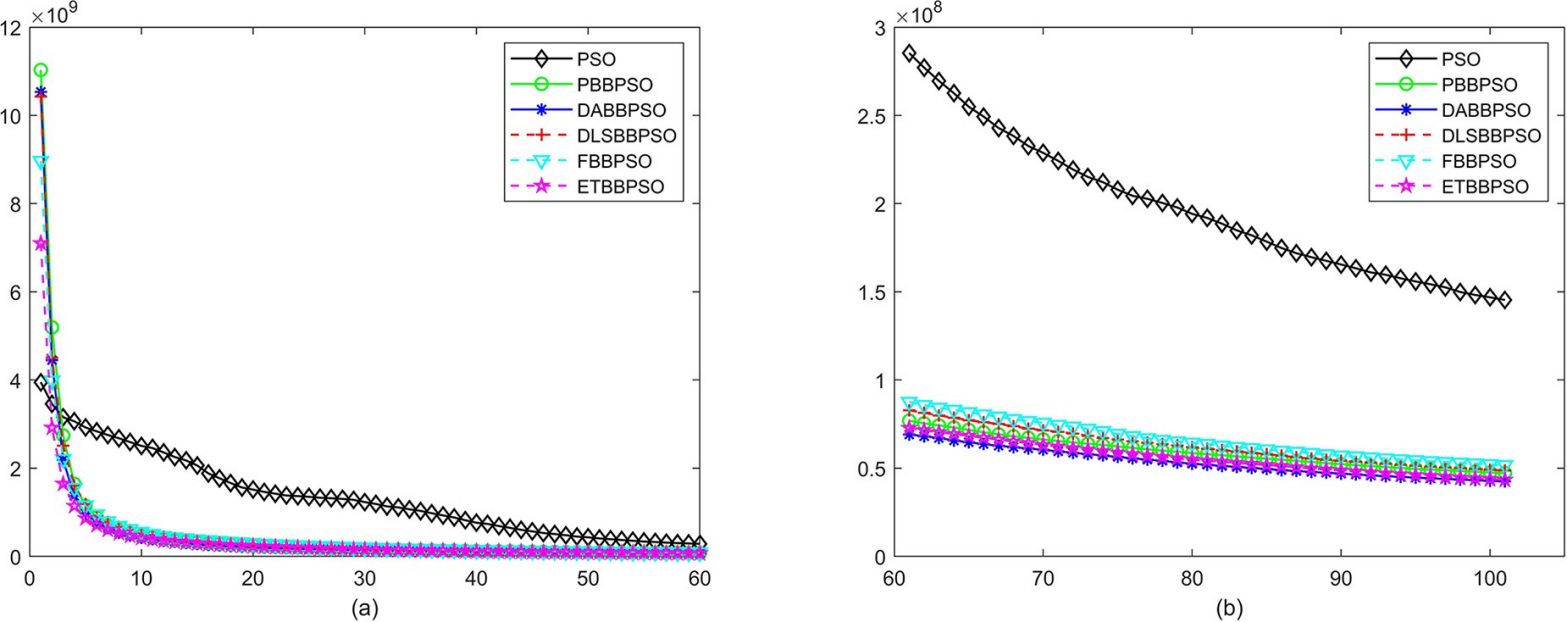
Comparison of convergence speed between PSO, PBBPSO, DA-BBPSO, DLS-BBPSO, FBBPSO and ETBBPSO, *f*_1_, (a) iteration 0-6000, (b) iteration 6000-10000 the unit is 100 iteration.

**Fig 3:**
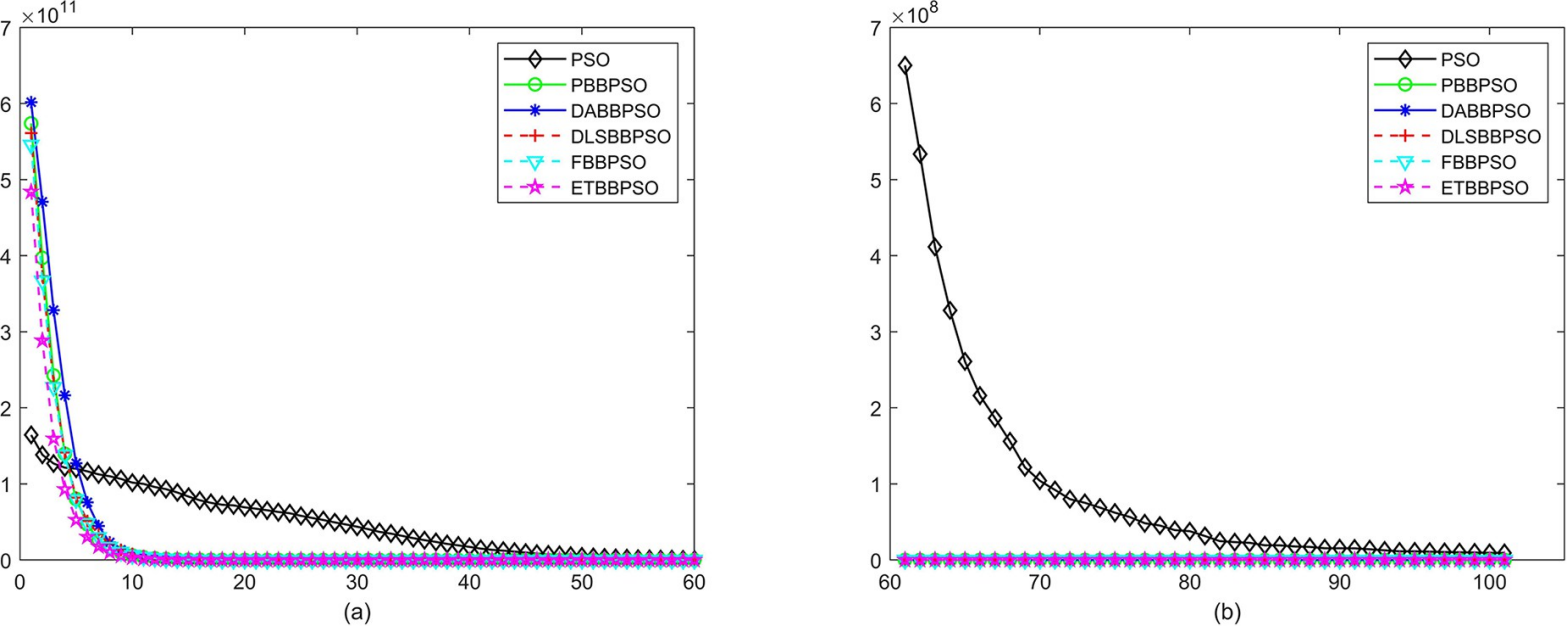
Comparison of convergence speed between PSO, PBBPSO, DA-BBPSO, DLS-BBPSO, FBBPSO and ETBBPSO, *f*_2_, (a) iteration 0-6000, (b) iteration 6000-10000 the unit is 100 iteration.

**Fig 4:**
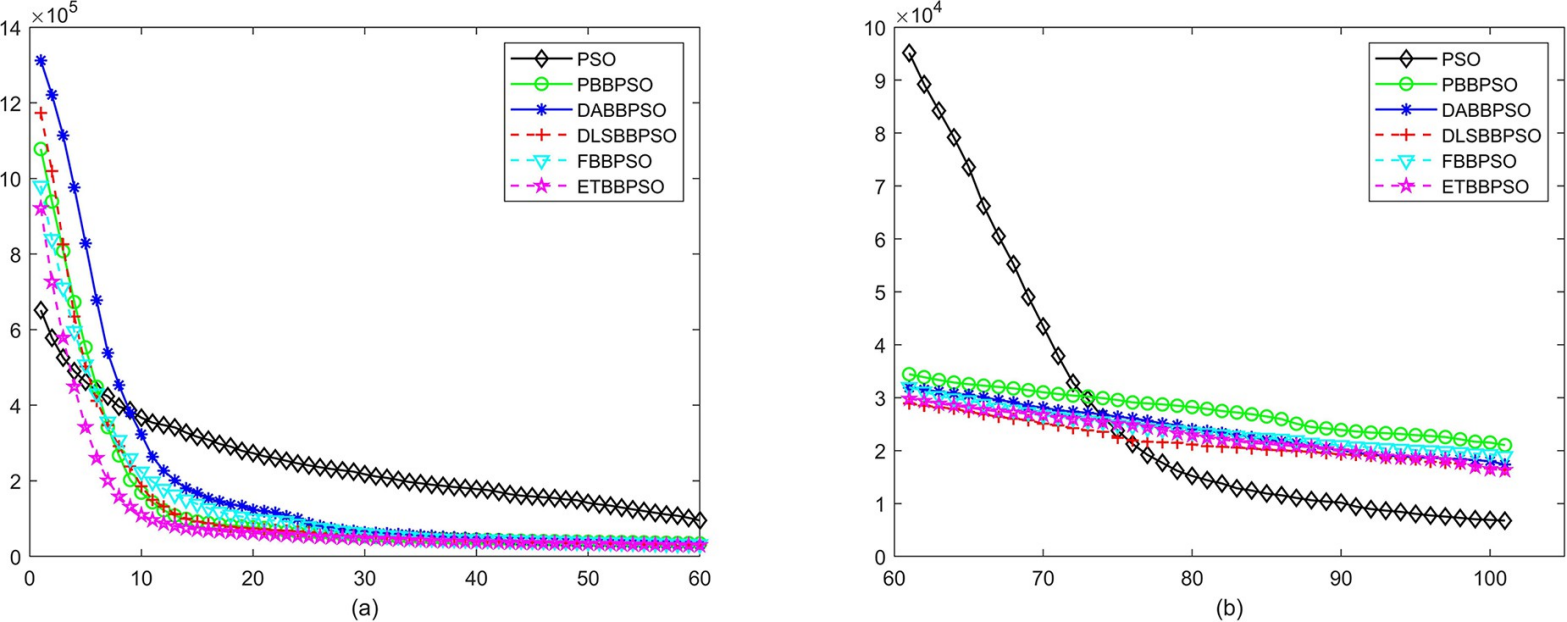
Comparison of convergence speed between PSO, PBBPSO, DA-BBPSO, DLS-BBPSO, FBBPSO and ETBBPSO, *f*_3_, (a) iteration 0-6000, (b) iteration 6000-10000 the unit is 100 iteration.

**Fig 5:**
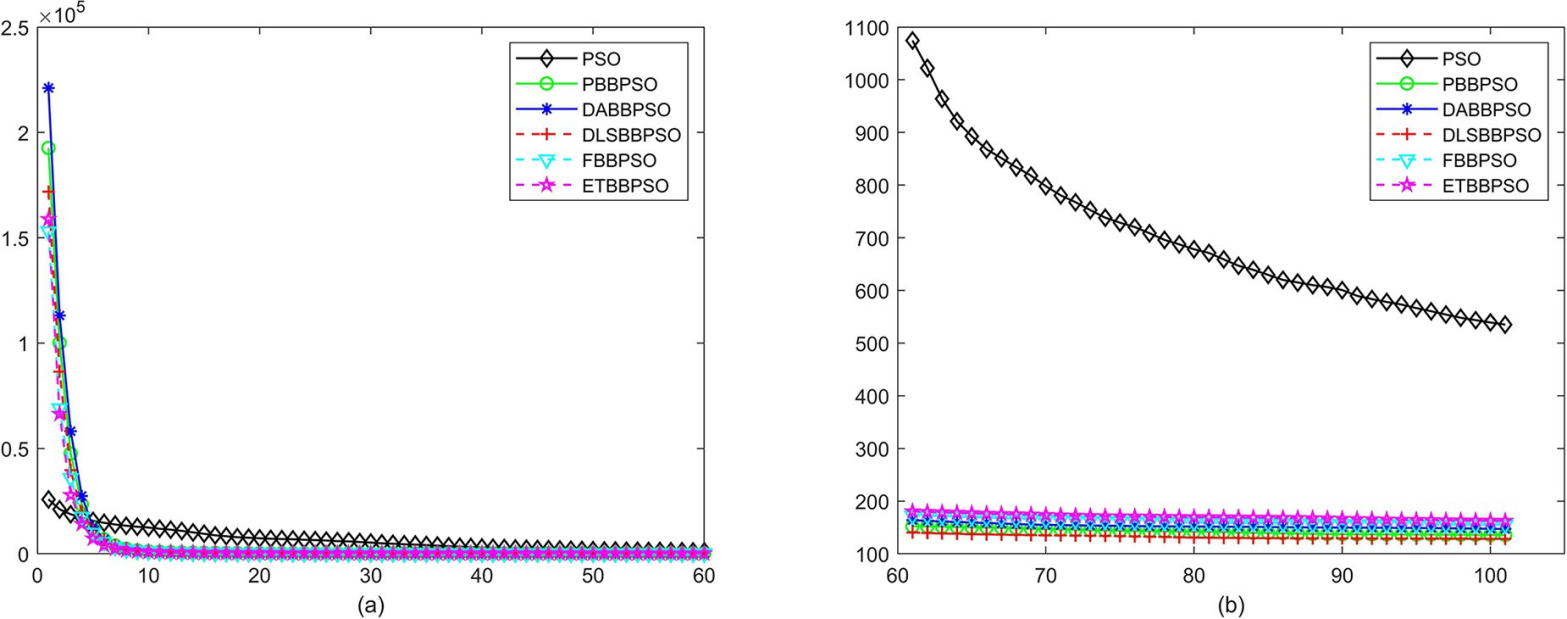
Comparison of convergence speed between PSO, PBBPSO, DA-BBPSO, DLS-BBPSO, FBBPSO and ETBBPSO, *f*_4_, (a) iteration 0-6000, (b) iteration 6000-10000 the unit is 100 iteration.

**Fig 6:**
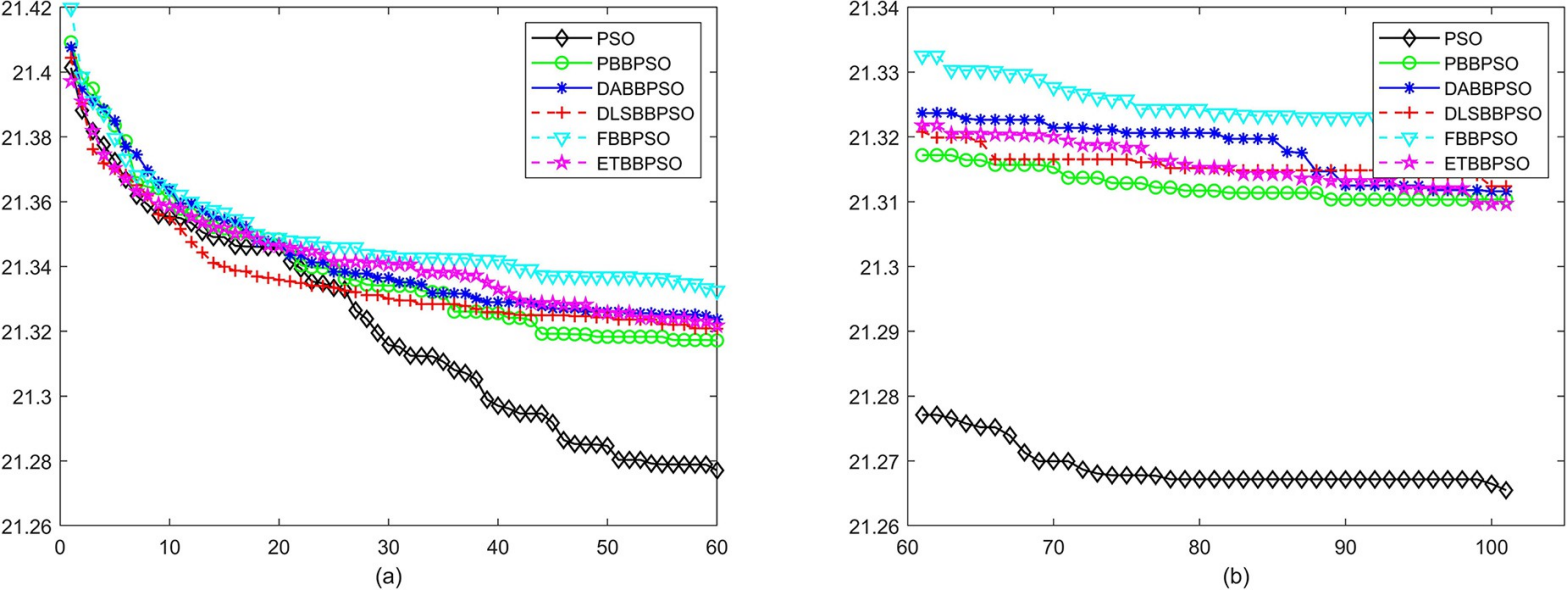
Comparison of convergence speed between PSO, PBBPSO, DA-BBPSO, DLS-BBPSO, FBBPSO and ETBBPSO, *f*_5_, (a) iteration 0-6000, (b) iteration 6000-10000 the unit is 100 iteration.

**Fig 7:**
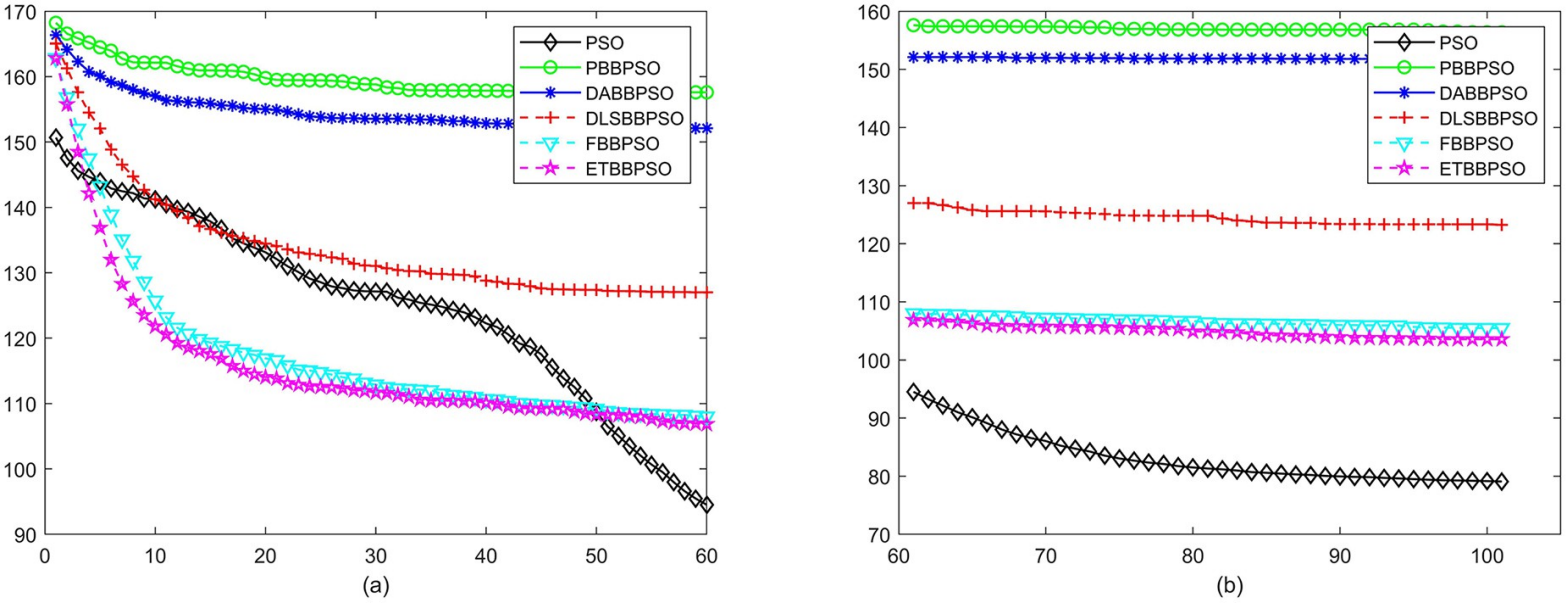
Comparison of convergence speed between PSO, PBBPSO, DA-BBPSO, DLS-BBPSO, FBBPSO and ETBBPSO, *f*_6_, (a) iteration 0-6000, (b) iteration 6000-10000 the unit is 100 iteration.

**Fig 8:**
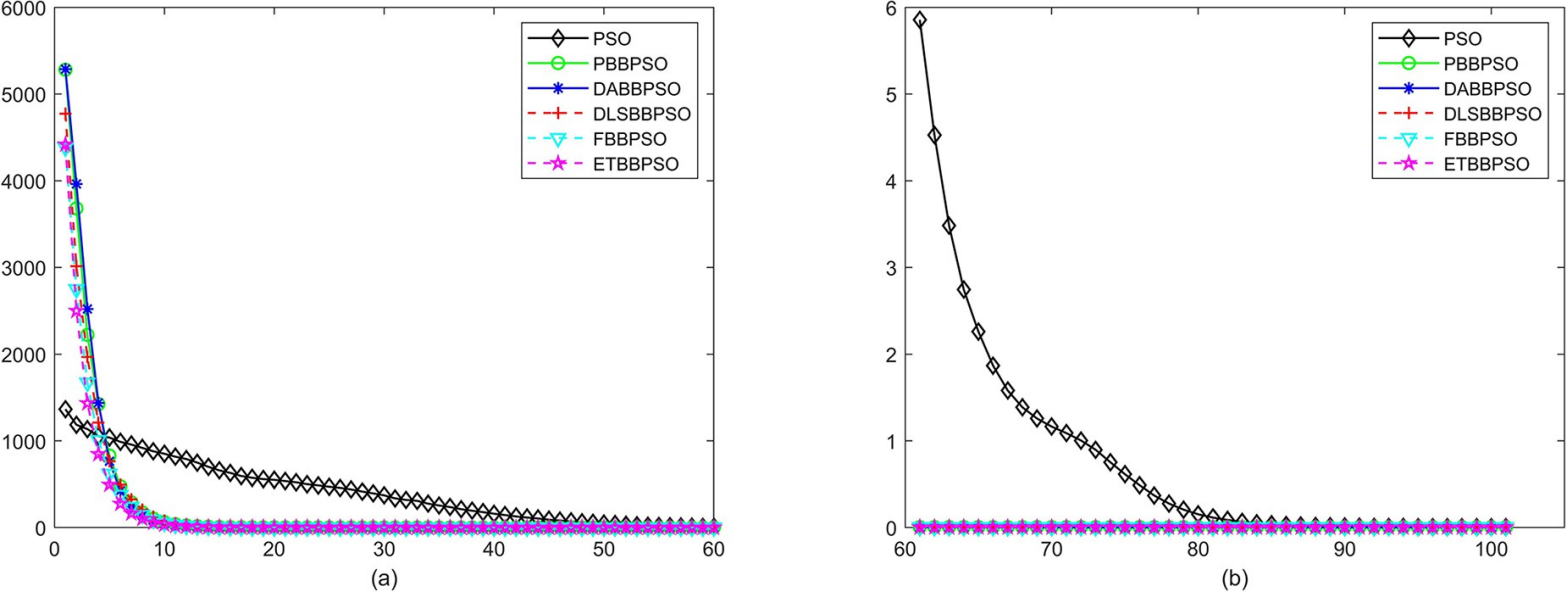
Comparison of convergence speed between PSO, PBBPSO, DA-BBPSO, DLS-BBPSO, FBBPSO and ETBBPSO, *f*_7_, (a) iteration 0-6000, (b) iteration 6000-10000 the unit is 100 iteration.

**Fig 9:**
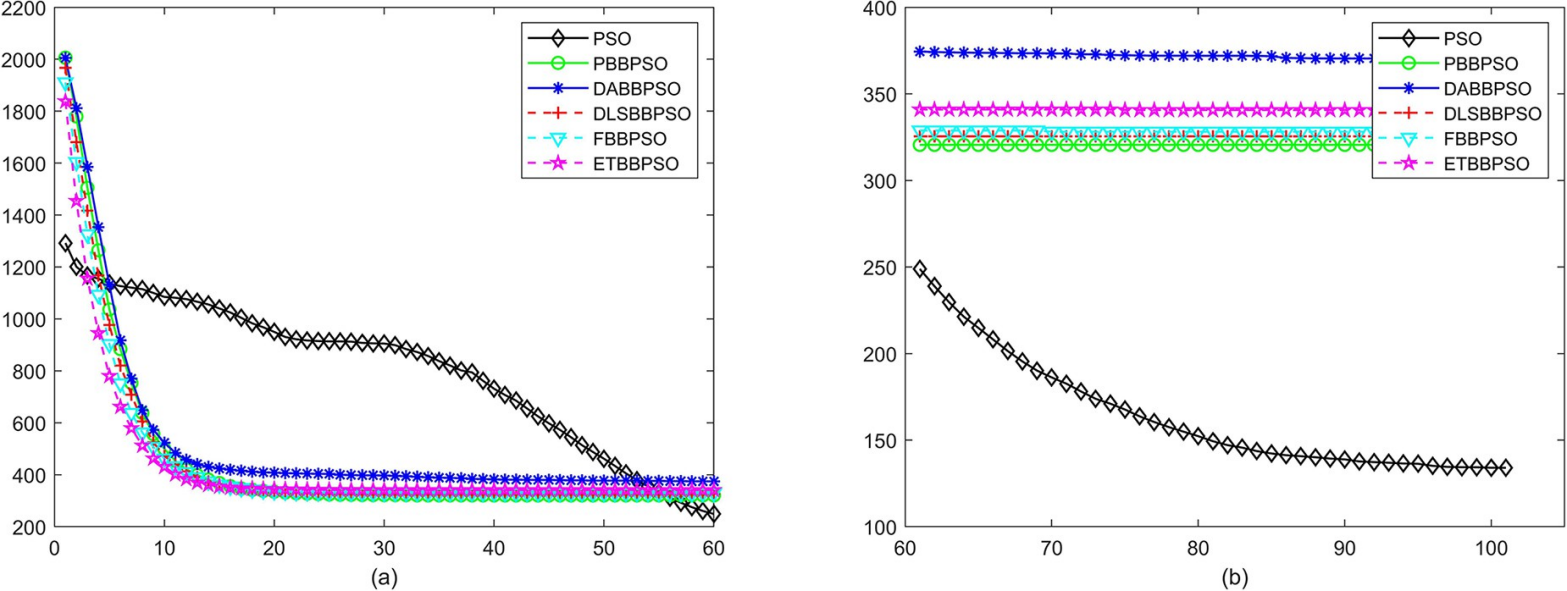
Comparison of convergence speed between PSO, PBBPSO, DA-BBPSO, DLS-BBPSO, FBBPSO and ETBBPSO, *f*_8_, (a) iteration 0-6000, (b) iteration 6000-10000 the unit is 100 iteration.

**Fig 10:**
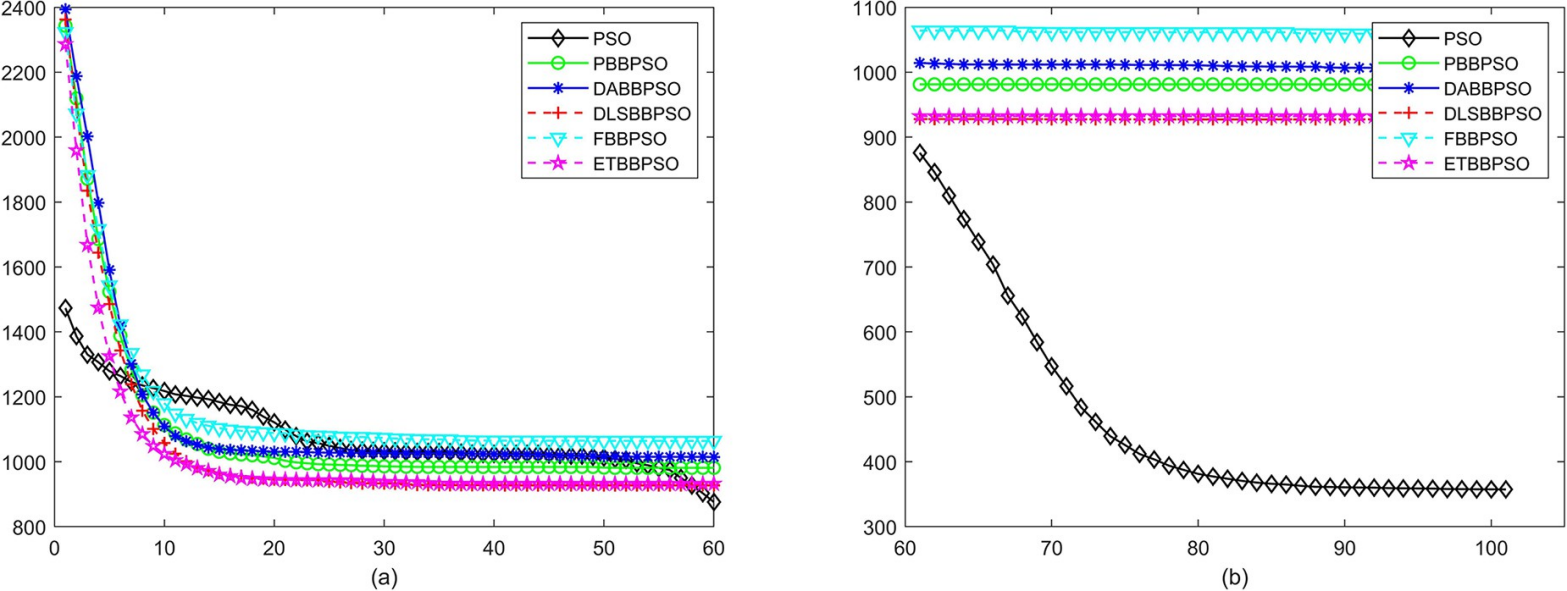
Comparison of convergence speed between PSO, PBBPSO, DA-BBPSO, DLS-BBPSO, FBBPSO and ETBBPSO, *f*_9_, (a) iteration 0-6000, (b) iteration 6000-10000 the unit is 100 iteration.

**Fig 11:**
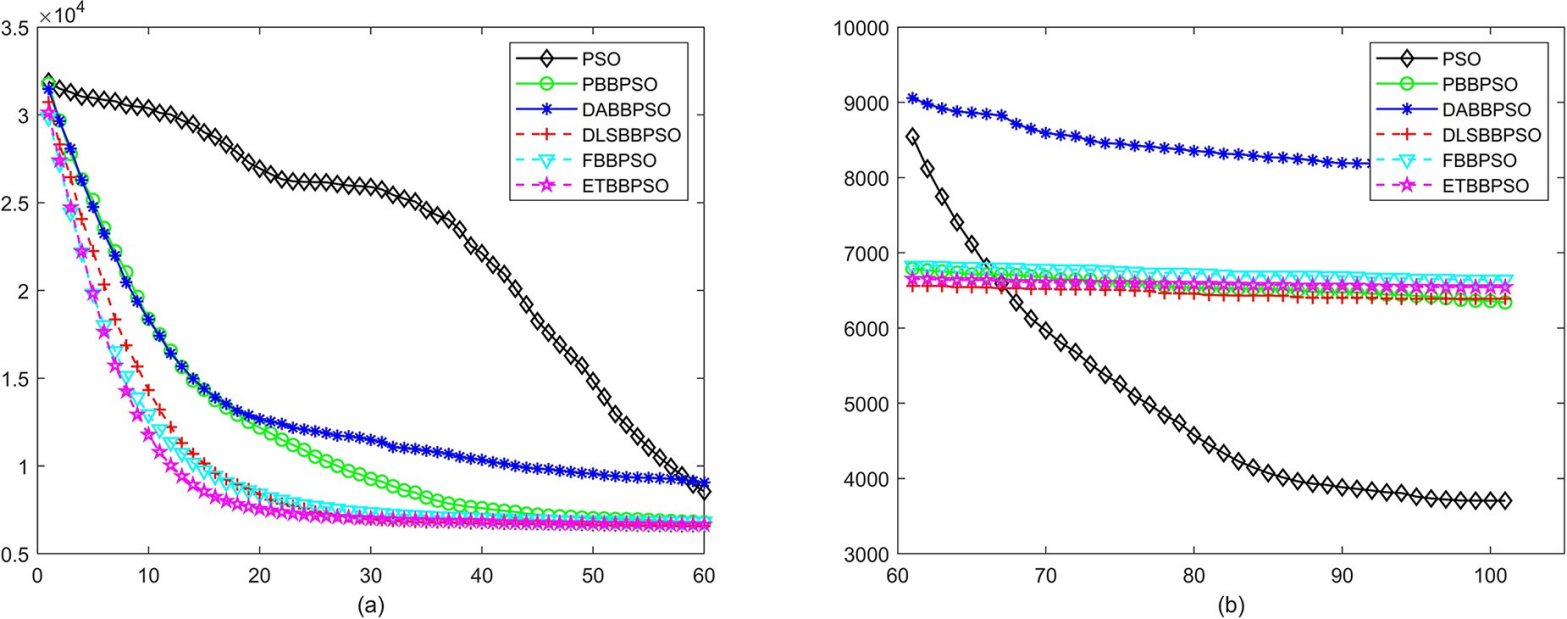
Comparison of convergence speed between PSO, PBBPSO, DA-BBPSO, DLS-BBPSO, FBBPSO and ETBBPSO, *f*_10_, (a) iteration 0-6000, (b) iteration 6000-10000 the unit is 100 iteration.

**Fig 12:**
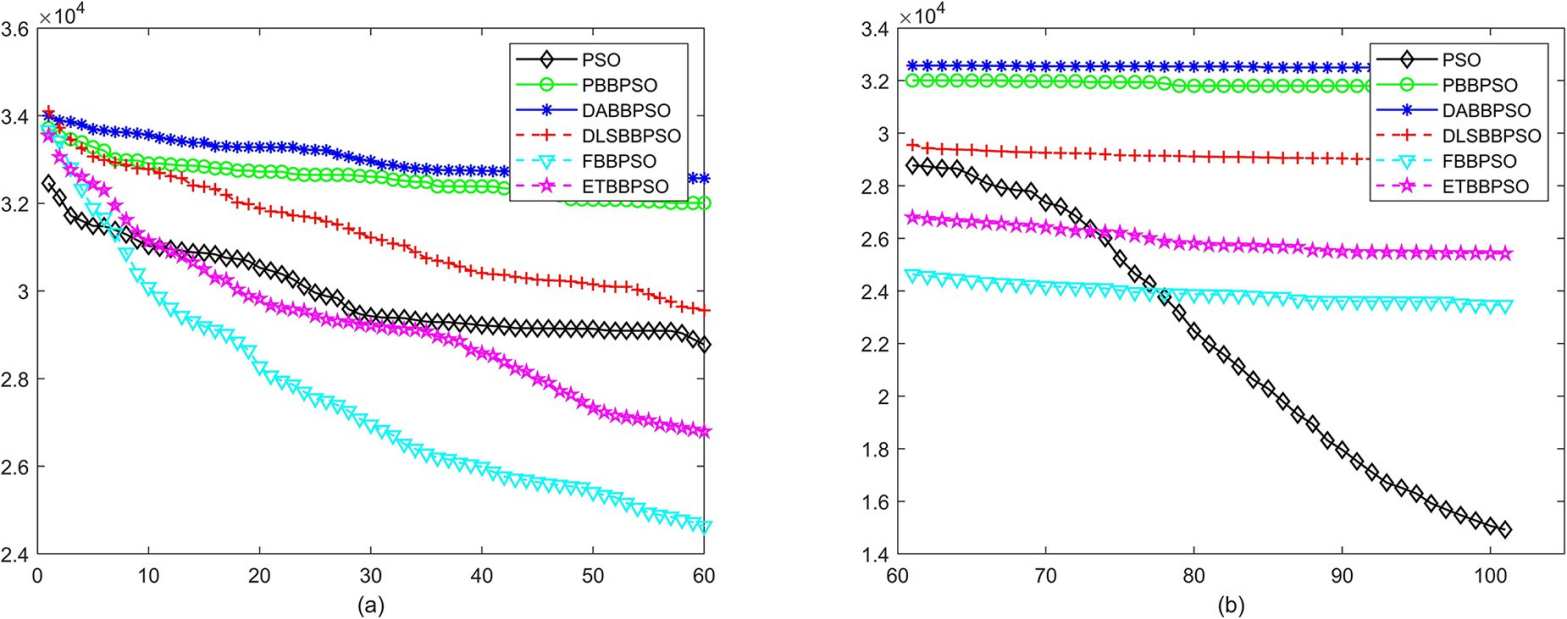
Comparison of convergence speed between PSO, PBBPSO, DA-BBPSO, DLS-BBPSO, FBBPSO and ETBBPSO, *f*_11_, (a) iteration 0-6000, (b) iteration 6000-10000 the unit is 100 iteration.

**Fig 13:**
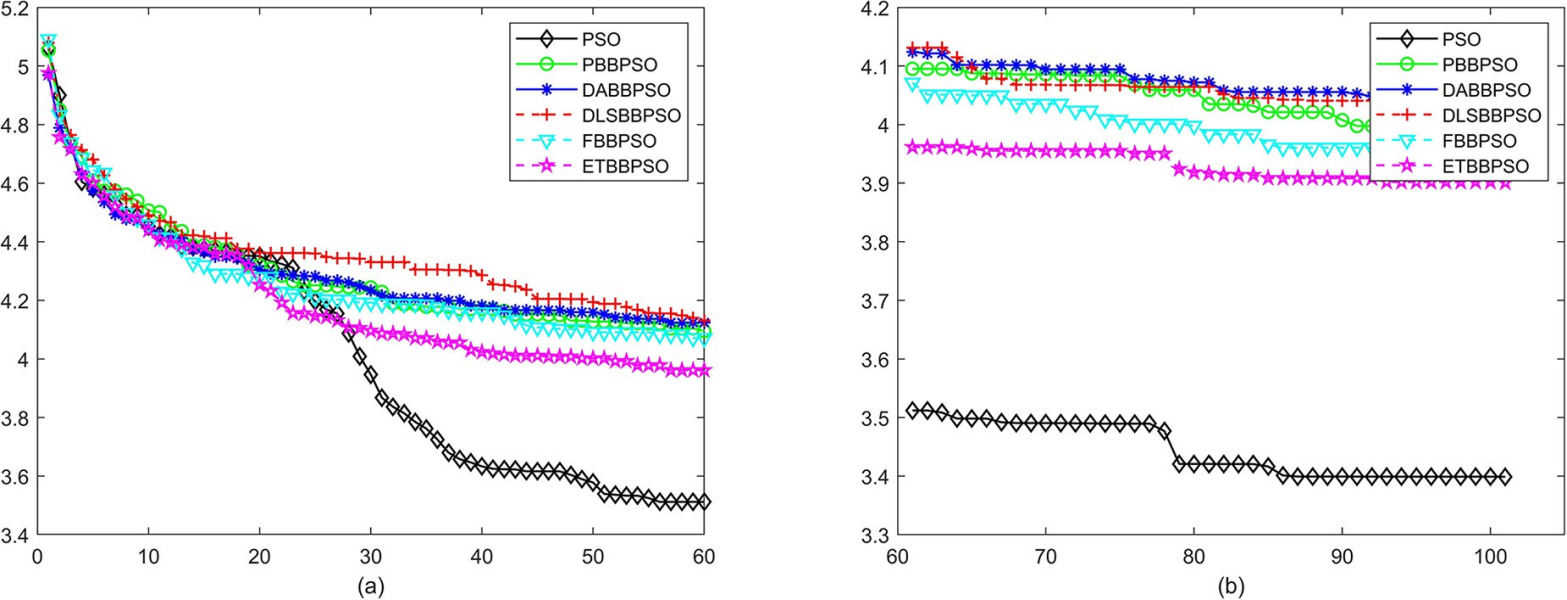
Comparison of convergence speed between PSO, PBBPSO, DA-BBPSO, DLS-BBPSO, FBBPSO and ETBBPSO, *f*_12_, (a) iteration 0-6000, (b) iteration 6000-10000 the unit is 100 iteration.

**Fig 14:**
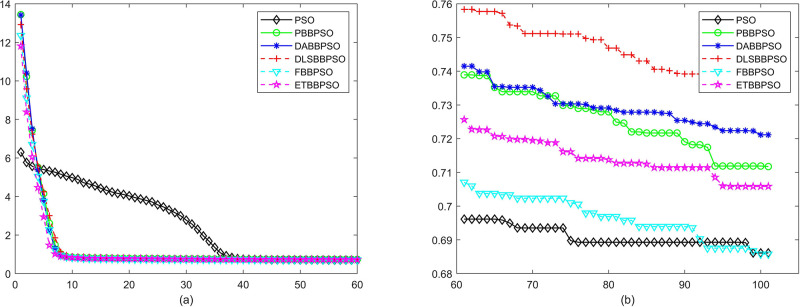
Comparison of convergence speed between PSO, PBBPSO, DA-BBPSO, DLS-BBPSO, FBBPSO and ETBBPSO, *f*_13_, (a) iteration 0-6000, (b) iteration 6000-10000, the unit is 100 iteration.

**Fig 15:**
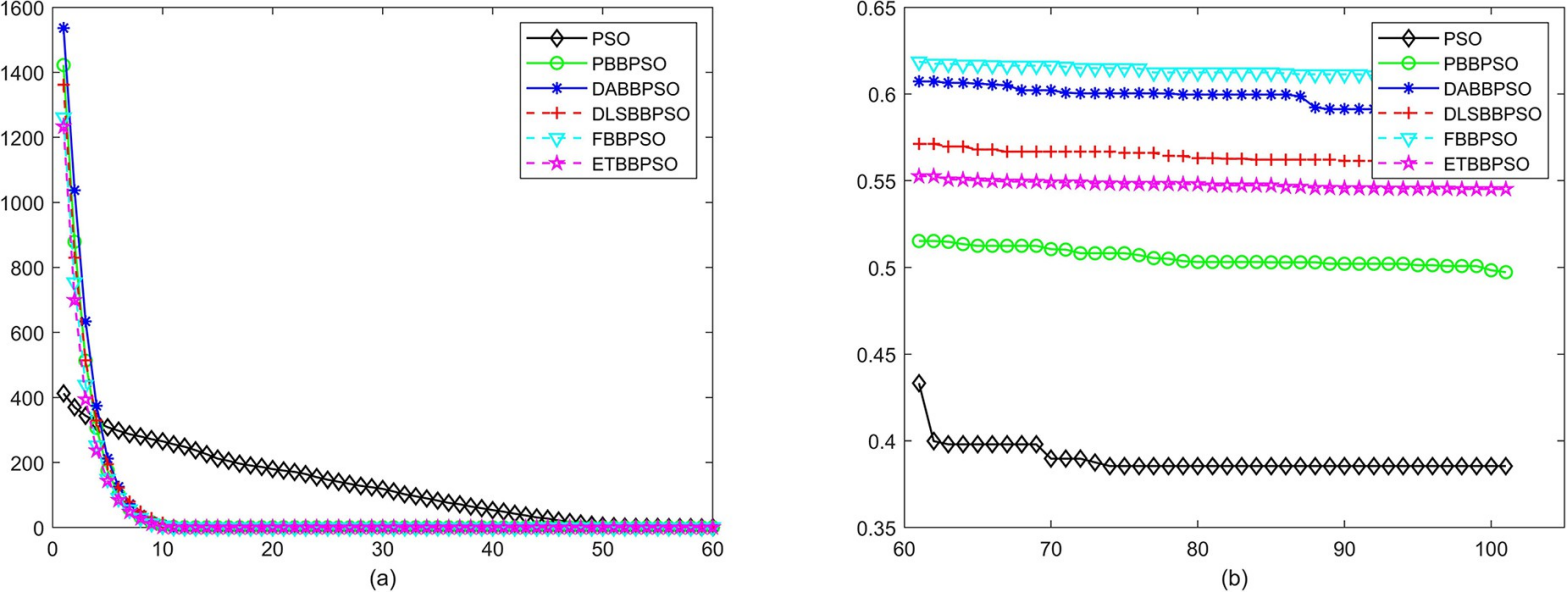
Comparison of convergence speed between PSO, PBBPSO, DA-BBPSO, DLS-BBPSO, FBBPSO and ETBBPSO, *f*_14_, (a) iteration 0-6000, (b) iteration 6000-10000 the unit is 100 iteration.

**Fig 16:**
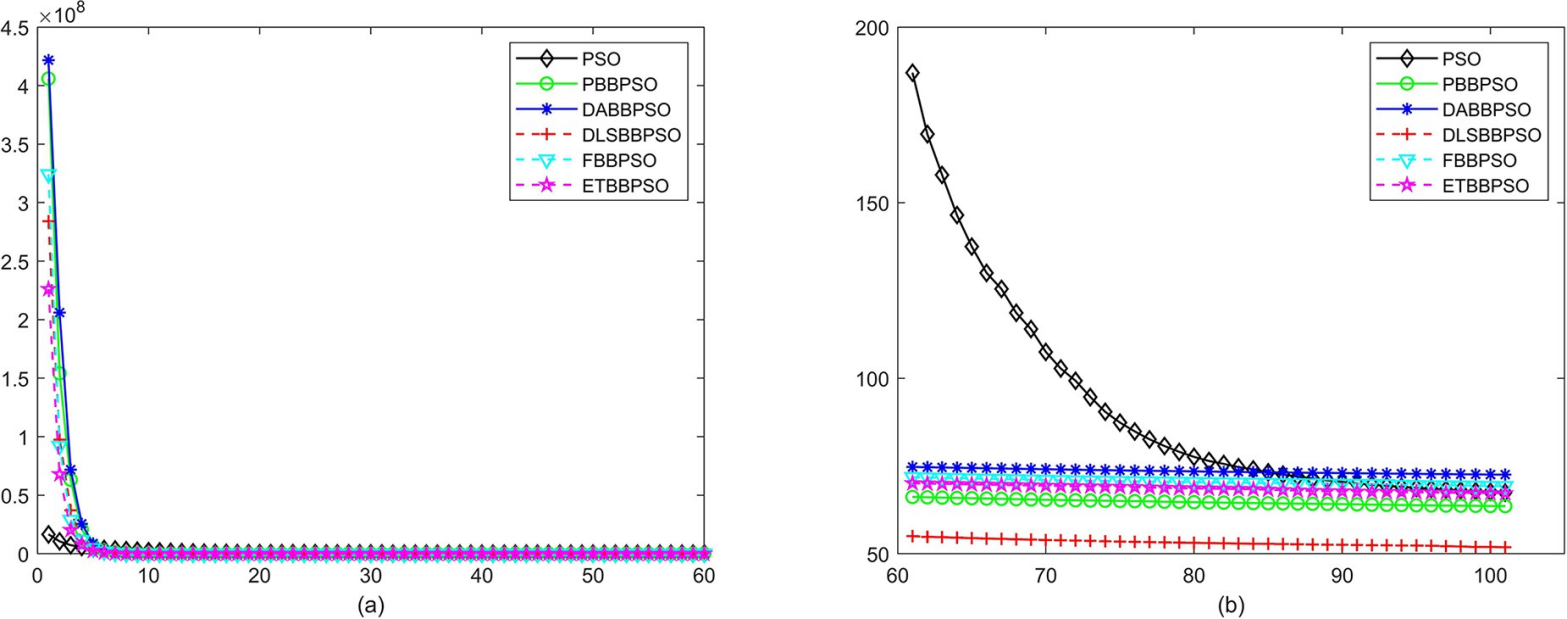
Comparison of convergence speed between PSO, PBBPSO, DA-BBPSO, DLS-BBPSO, FBBPSO and ETBBPSO, *f*_15_, (a) iteration 0-6000, (b) iteration 6000-10000 the unit is 100 iteration.

**Fig 17:**
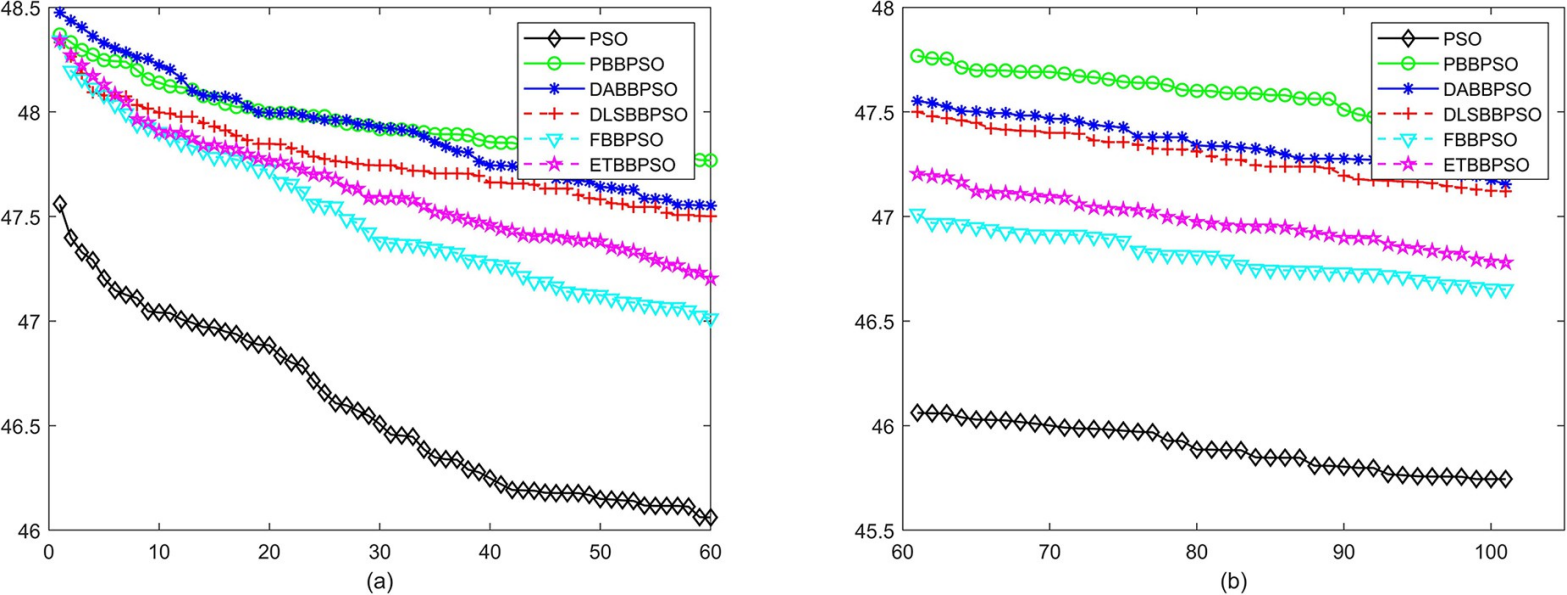
Comparison of convergence speed between PSO, PBBPSO, DA-BBPSO, DLS-BBPSO, FBBPSO and ETBBPSO, *f*_16_, (a) iteration 0-6000, (b) iteration 6000-10000 the unit is 100 iteration.

**Fig 18:**
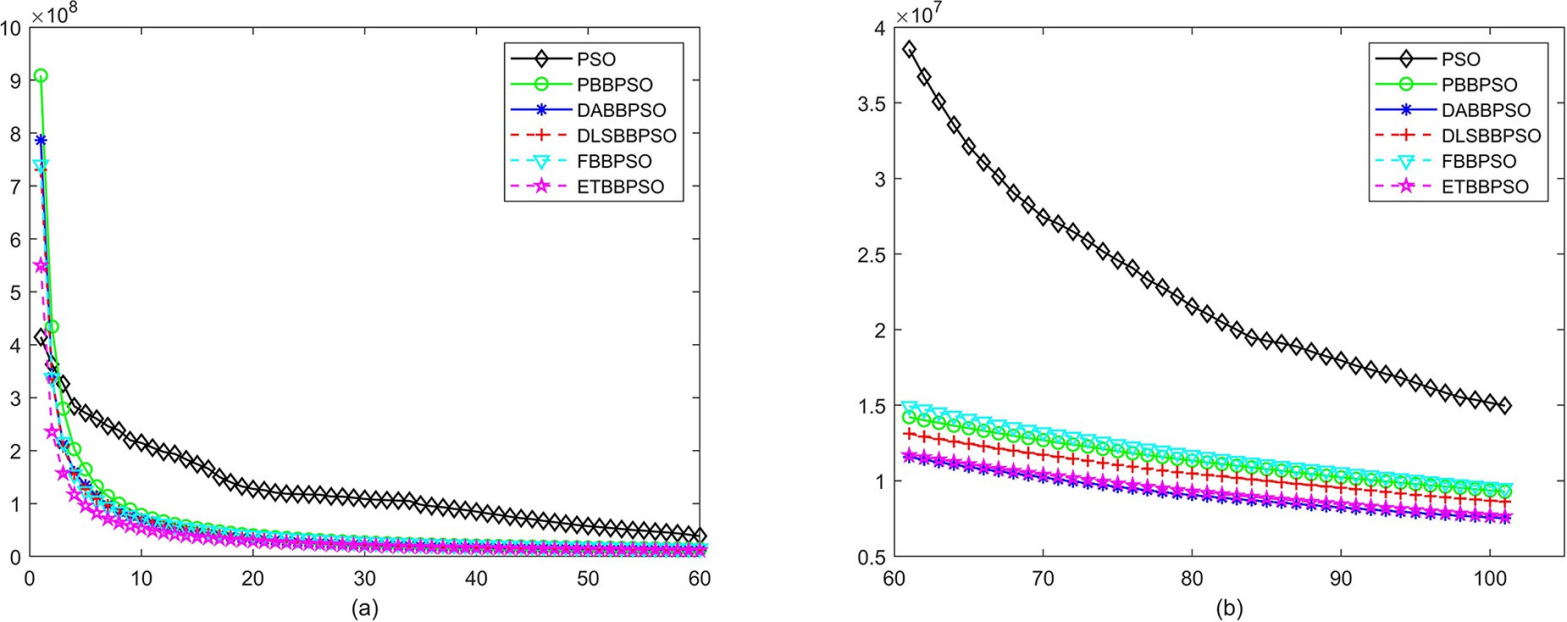
Comparison of convergence speed between PSO, PBBPSO, DA-BBPSO, DLS-BBPSO, FBBPSO and ETBBPSO, *f*_17_, (a) iteration 0-6000, (b) iteration 6000-10000 the unit is 100 iteration.

**Fig 19:**
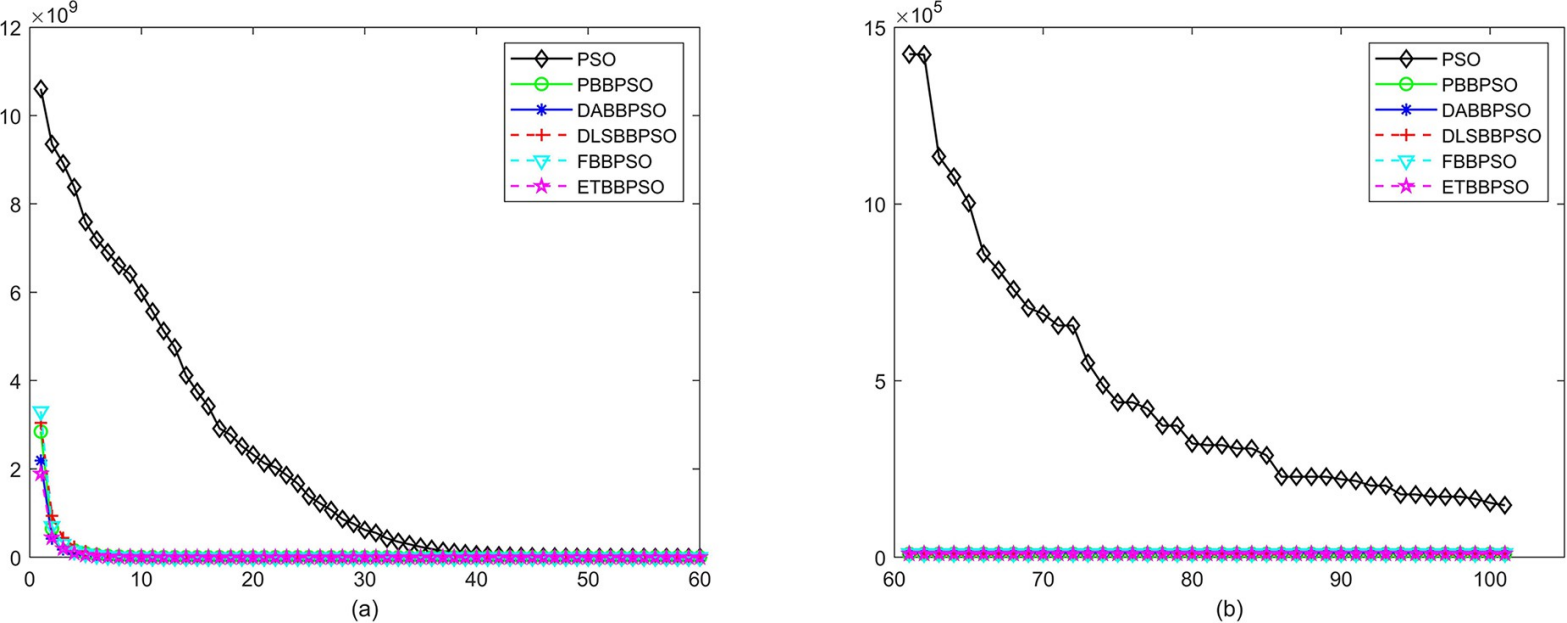
Comparison of convergence speed between PSO, PBBPSO, DA-BBPSO, DLS-BBPSO, FBBPSO and ETBBPSO,*f*_18_, (a) iteration 0-6000, (b) iteration 6000-10000 the unit is 100 iteration.

**Fig 20:**
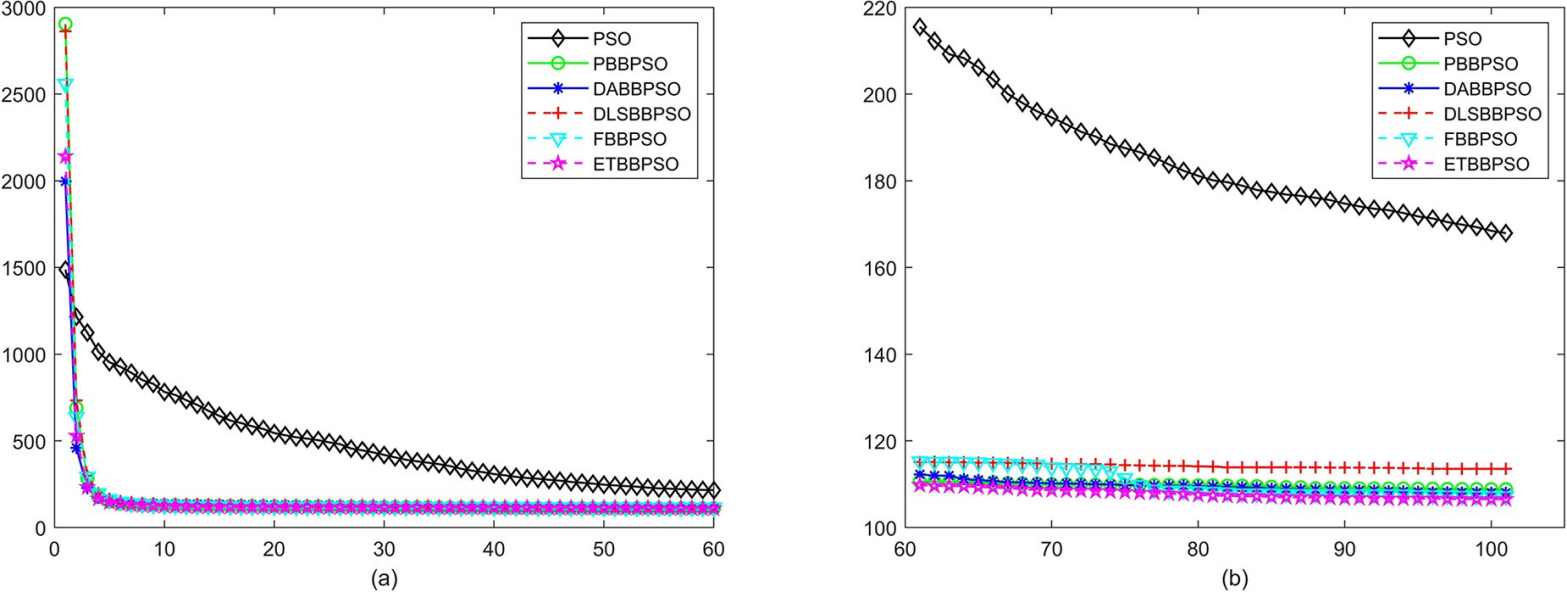
Comparison of convergence speed between PSO, PBBPSO, DA-BBPSO, DLS-BBPSO, FBBPSO and ETBBPSO, *f*_19_, (a) iteration 0-6000, (b) iteration 6000-10000 the unit is 100 iteration.

**Fig 21:**
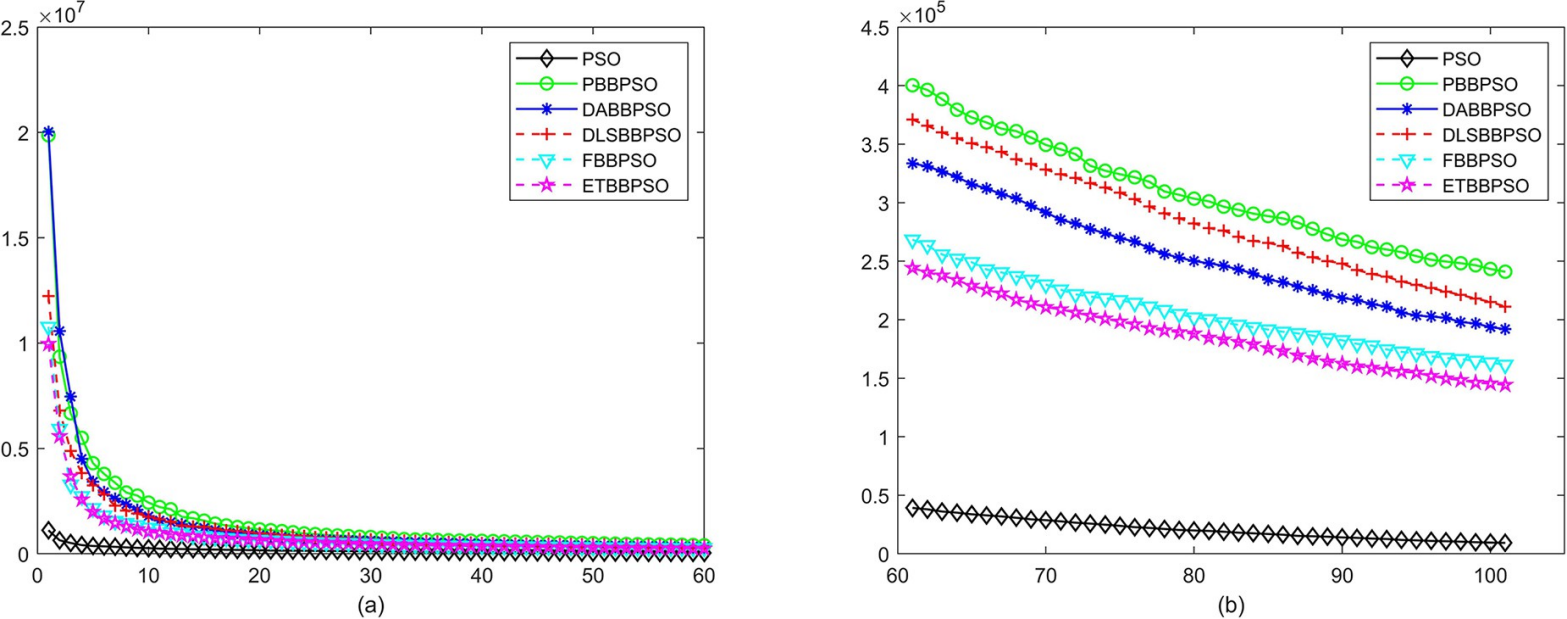
Comparison of convergence speed between PSO, PBBPSO, DA-BBPSO, DLS-BBPSO, FBBPSO and ETBBPSO, *f*_20_, (a) iteration 0-6000, (b) iteration 6000-10000 the unit is 100 iteration.

**Fig 22:**
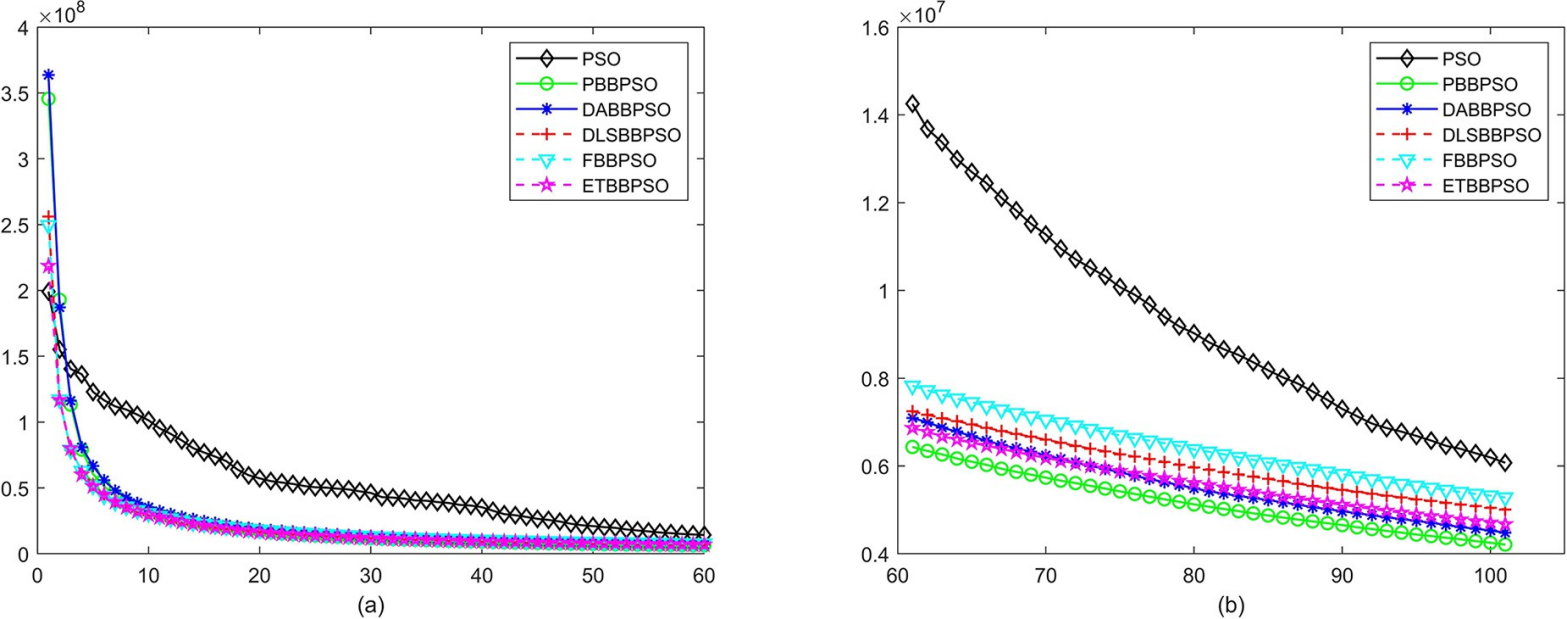
Comparison of convergence speed between PSO, PBBPSO, DA-BBPSO, DLS-BBPSO, FBBPSO and ETBBPSO, *f*_21_, (a) iteration 0-6000, (b) iteration 6000-10000 the unit is 100 iteration.

**Fig 23:**
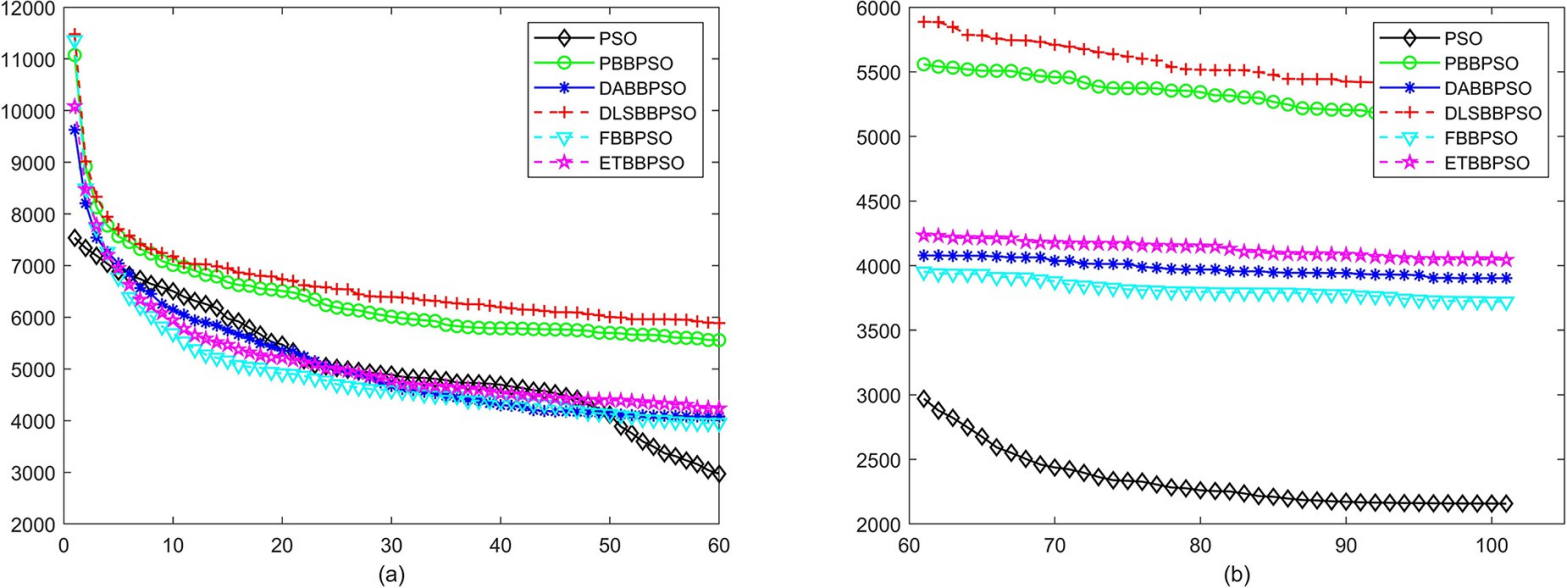
Comparison of convergence speed between PSO, PBBPSO, DA-BBPSO, DLS-BBPSO, FBBPSO and ETBBPSO, *f*_22_, (a) iteration 0-6000, (b) iteration 6000-10000 the unit is 100 iteration.

**Fig 24:**
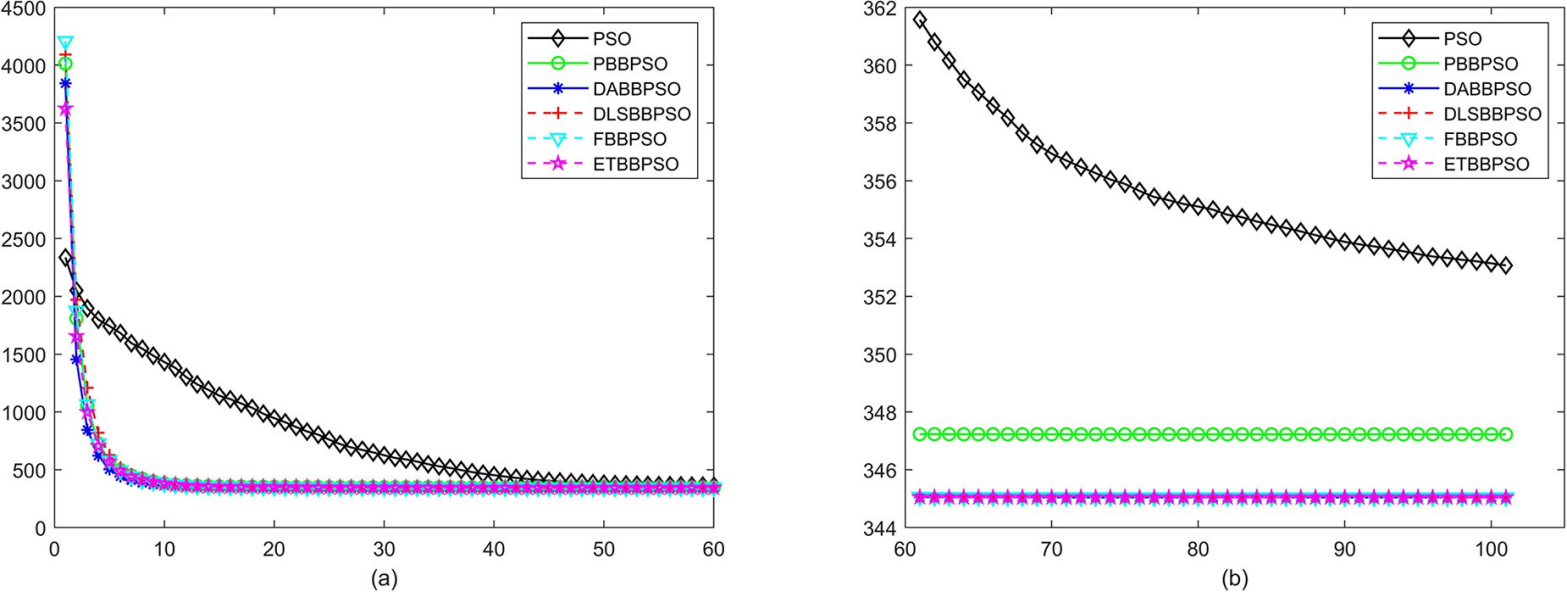
Comparison of convergence speed between PSO, PBBPSO, DA-BBPSO, DLS-BBPSO, FBBPSO and ETBBPSO, *f*_23_, (a) iteration 0-6000, (b) iteration 6000-10000 the unit is 100 iteration.

**Fig 25:**
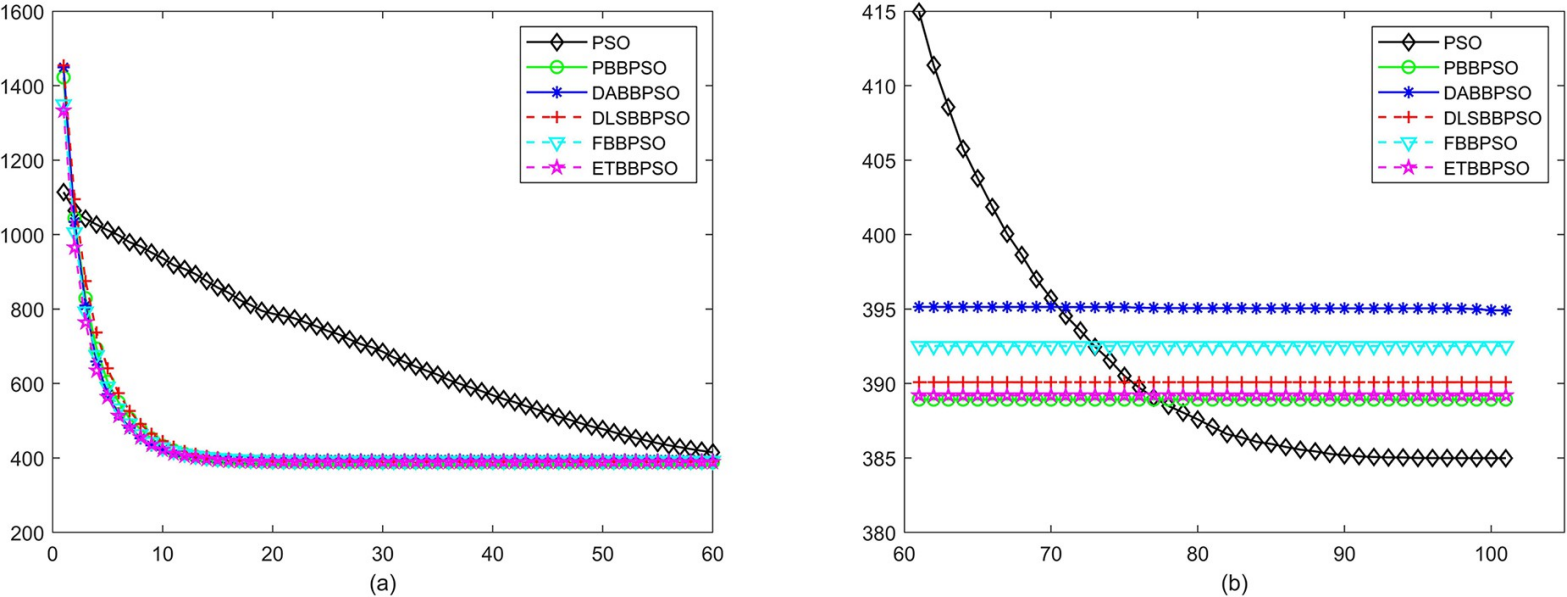
Comparison of convergence speed between PSO, PBBPSO, DA-BBPSO, DLS-BBPSO, FBBPSO and ETBBPSO, *f*_24_, (a) iteration 0-6000, (b) iteration 6000-10000 the unit is 100 iteration.

**Fig 26:**
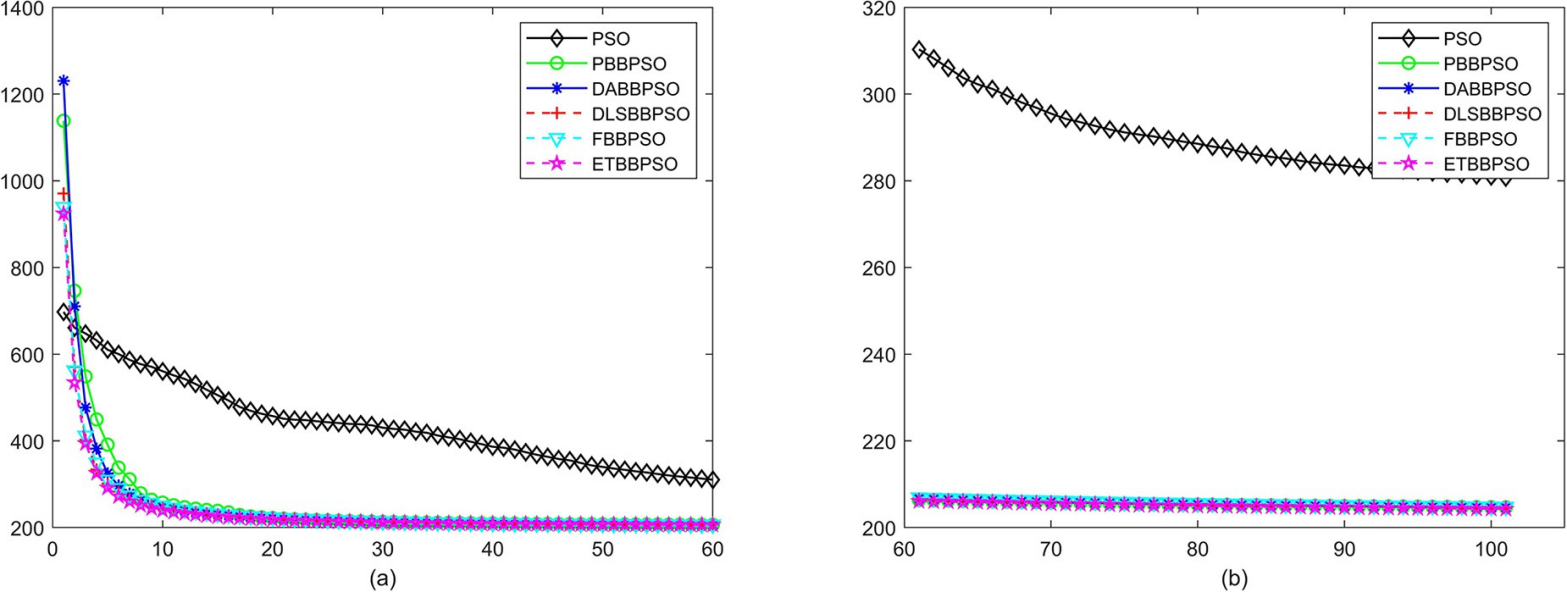
Comparison of convergence speed between PSO, PBBPSO, DA-BBPSO, DLS-BBPSO, FBBPSO and ETBBPSO, *f*_25_, (a) iteration 0-6000, (b) iteration 6000-10000 the unit is 100 iteration.

**Fig 27:**
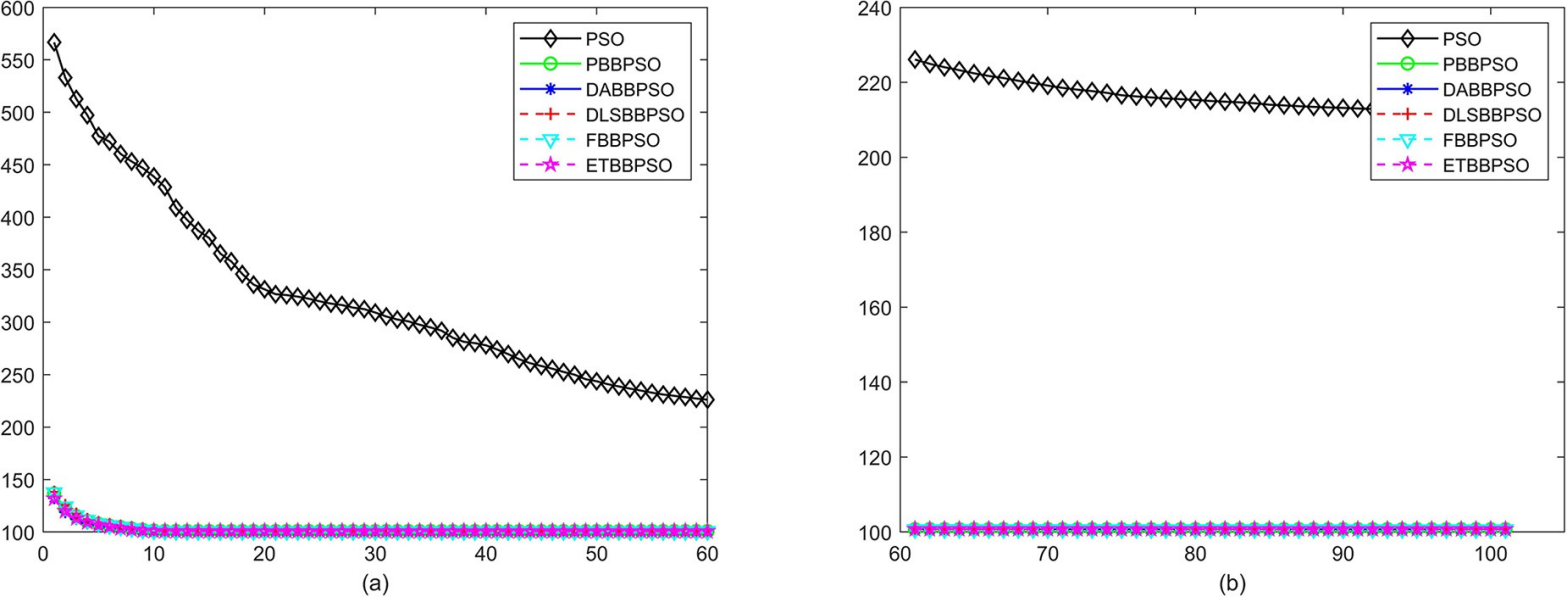
Comparison of convergence speed between PSO, PBBPSO, DA-BBPSO, DLS-BBPSO, FBBPSO and ETBBPSO, *f*_26_, (a) iteration 0-6000, (b) iteration 6000-10000 the unit is 100 iteration.

**Fig 28:**
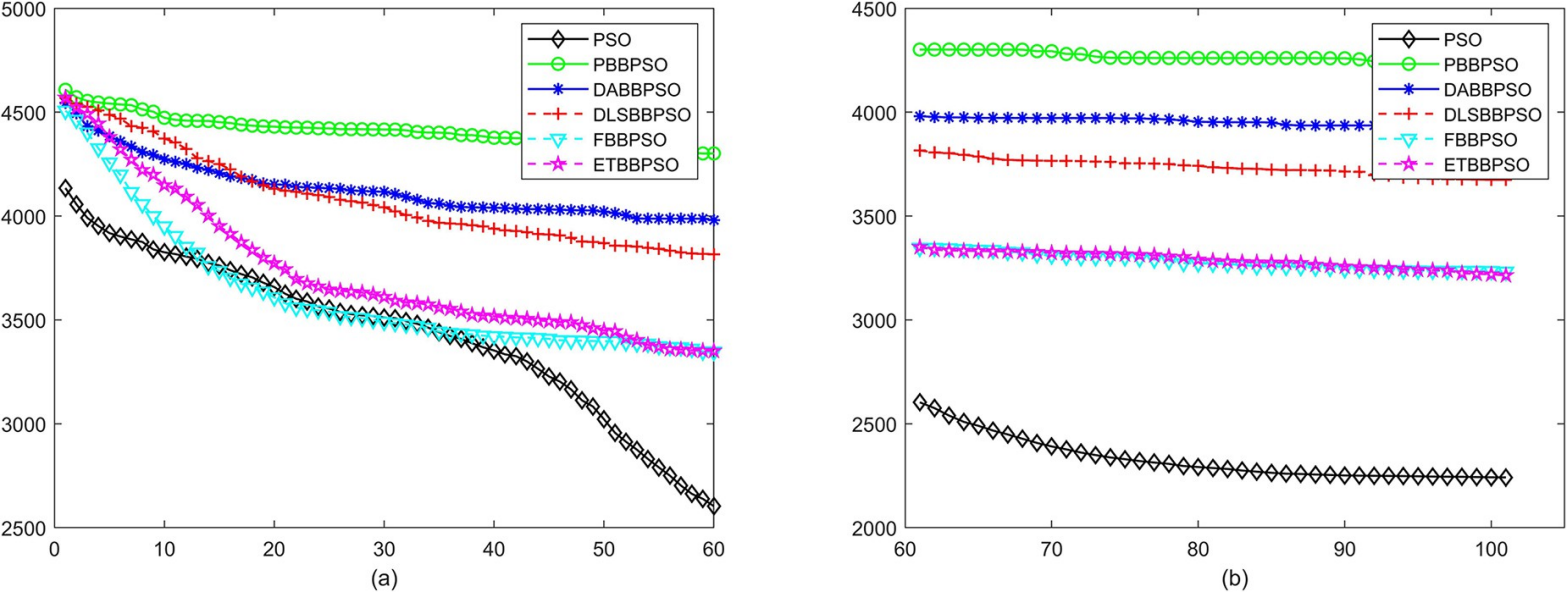
Comparison of convergence speed between PSO, PBBPSO, DA-BBPSO, DLS-BBPSO, FBBPSO and ETBBPSO, *f*_27_, (a) iteration 0-6000, (b) iteration 6000-10000 the unit is 100 iteration.

**Fig 29:**
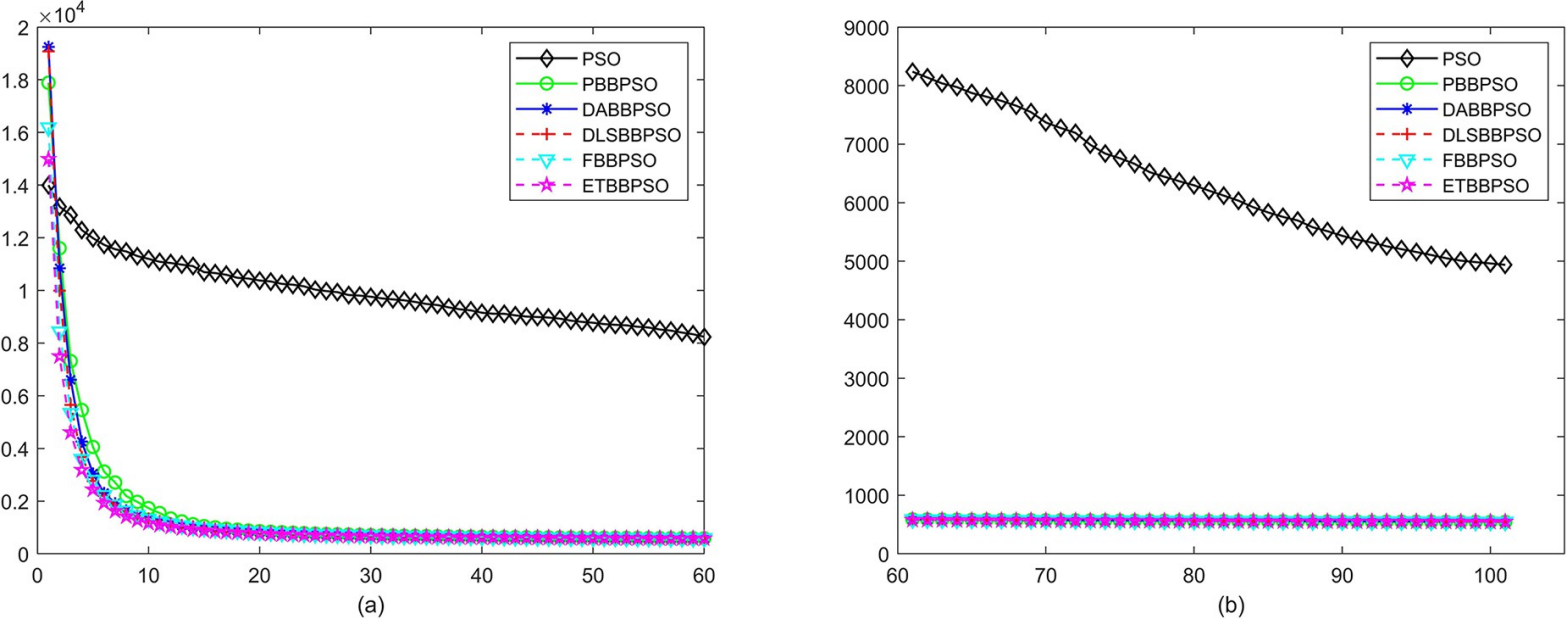
Comparison of convergence speed between PSO, PBBPSO, DA-BBPSO, DLS-BBPSO, FBBPSO and ETBBPSO, *f*_28_, (a) iteration 0-6000, (b) iteration 6000-10000 the unit is 100 iteration.

**Fig 30:**
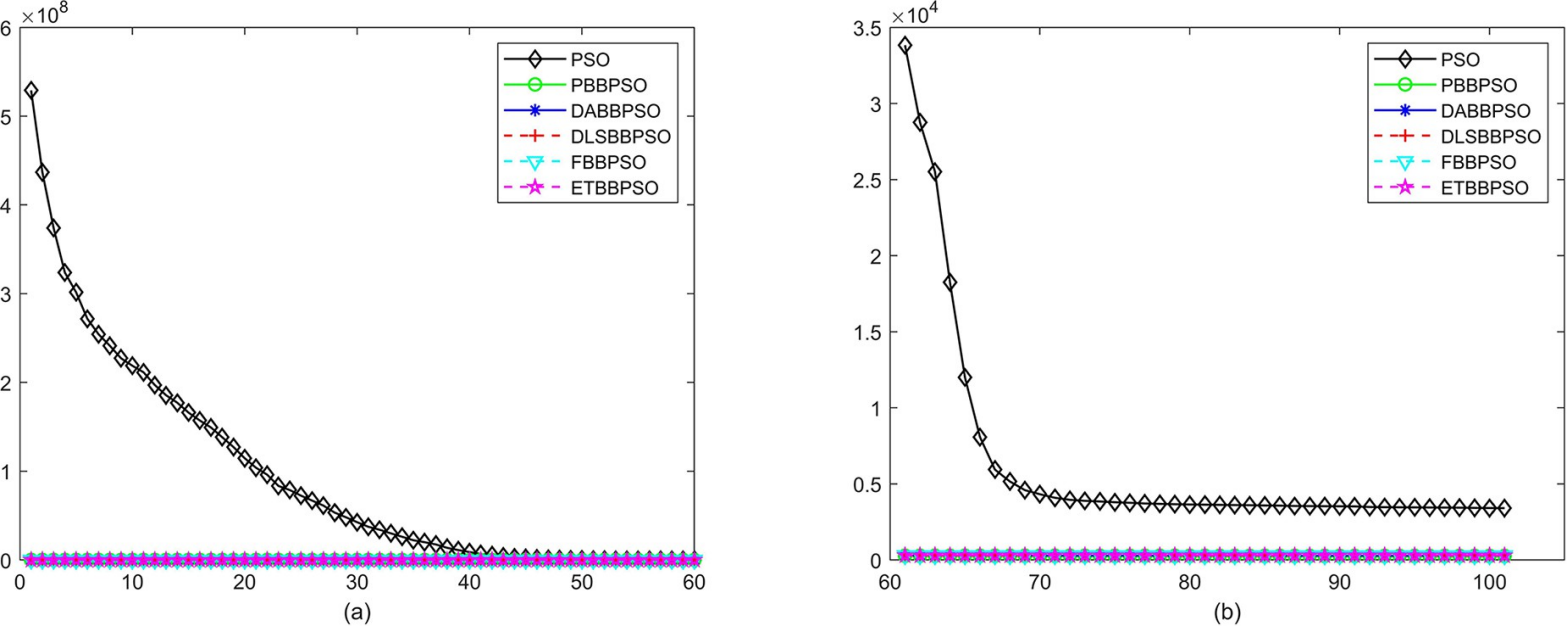
Comparison of convergence speed between PSO, PBBPSO, DA-BBPSO, DLS-BBPSO, FBBPSO and ETBBPSO, *f*_29_, (a) iteration 0-6000, (b) iteration 6000-10000 the unit is 100 iteration.

**Fig 31:**
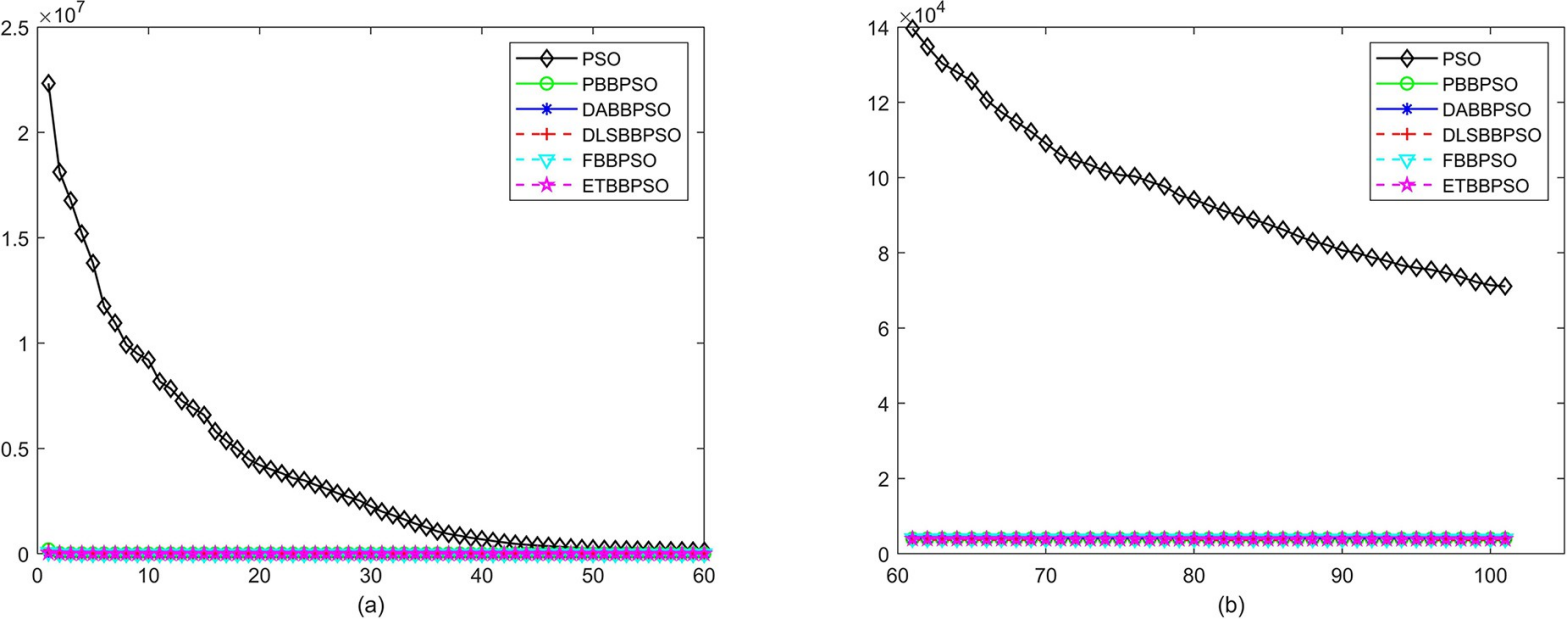
Comparison of convergence speed between PSO, PBBPSO, DA-BBPSO, DLS-BBPSO, FBBPSO and ETBBPSO, *f*_30_, (a) iteration 0-6000, (b) iteration 6000-10000 the unit is 100 iteration.

#### Discussion

A ranking competition is designed for every test function. The algorithm presents the best results will get 1 point, presents the second-best results will get 2 point, presents the third-best results will get 3 point, presents the forth-best results will get 4 point, presents the fifth-best results will get 5 point, presents the worst results will get 6 point. The mean ranking results are show in the bottom of Table 3. ETBBPSO shows the best results in the 100-dimension test. This is mainly because DPG is able to divide the particle swarm into different orbits. The whole partitioning process is self-controlled by the algorithm and does not require any parameters. Then, PTO enhances the local search ability of particle swarm while taking into account the global search ability, making it more possible for the particle swarm to escape from the local optimum.

### Experiments with CEC2020

The standard BBPSO and the ETBBPSO are tested with the CEC2020 benchmark functions. Parameters are shown in Table 4. Experimental results are shown in Table 5. CE is defined as |*GlobalBestValue*−*TheoreticallyMinimum*|. The ETBBPSO scored 4 firsts and 4 seconds in 10 test functions, shown great performance in this experiments.

**Table 5:** Experimental Results with CEC2020, CEs of BBPSO and ETBBPSO. Mean is the mean valut from 31 independent runs, STD is the standard deviation of the 31 runs.

Function Number	Data Tpye	FBBPSO	BBPSO	ETBBPSO
*f*_1_	Mean	3.002E+04	1.278E+04	1.778E+04
	Std	3.915E+04	2.346E+04	3.322E+04
	Rank	3	1	2
*f*_2_	Mean	5.768E+02	6.037E+02	5.568E+02
	Std	2.124E+02	2.718E+02	2.086E+02
	Rank	2	3	1
*f*_3_	Mean	4.746E+01	4.553E+01	4.348E+01
	Std	9.161E+00	1.028E+01	1.141E+01
	Rank	3	2	1
*f*_4_	Mean	2.238E+00	2.506E+00	2.473E+00
	Std	1.021E-00	9.659E-01	8.925E-01
	Rank	1	3	2
*f*_5_	Mean	9.121E+04	8.048E+04	7.323E+04
	Std	8.335E+04	7.829E+04	7.657E+04
	Rank	3	2	1
*f*_6_	Mean	1.176E+01	2.218E+01	2.948E+01
	Std	1.335E+01	3.763E+01	4.494E+01
	Rank	1	2	3
*f*_7_	Mean	4.601E+04	3.929E+04	4.205E+04
	Std	3.750E+04	2.595E+04	4.416E+04
	Rank	3	1	2
*f*_8_	Mean	1.429E+03	1.219E+03	8.123E+02
	Std	1.217E+03	1.085E+03	1.013E+03
	Rank	3	2	1
*f*_9_	Mean	4.614E+02	4.725E+02	4.623E+02
	Std	2.869E+01	2.237E+01	2.611E+01
	Rank	1	3	2
*f*_10_	Mean	4.363E+02	4.317E+02	4.387E+02
	Std	3.185E+01	3.263E+01	3.014E+01
	Rank	2	1	3
Average Rank		2.2	2	1.8

## Conclusions

In this paper, a novel electronic transition-based bare bones particle swarm optimization (ETBBPSO) algorithm is proposed for high dimensional optimization problems. A dynamic particle grouping (DPG) strategy and a particle transition operator (PTO) collaborate to find the global optimal solution for high dimensional optimization problems. The DPG is mainly used to assign particles to different orbits, with a variable number of orbits and a variable number of particles per orbit. The PTO is used to combine the best and the second-best orbits. Particles that belong to the second-best orbits will transit to the best orbit to enhance the local search ability of the best orbit. A set of comprehensive experiments are designed to verify the optimization ability of ETBBPSO. Several famous population-based methods are used in the control group. Experimental results prove that ETBBPSO is able to present high precision results for high dimensional optimization problems.
